# Personizing the prediction of future susceptibility to a specific disease

**DOI:** 10.1371/journal.pone.0243127

**Published:** 2021-01-06

**Authors:** Kamal Taha, Ramana Davuluri, Paul Yoo, Jesse Spencer

**Affiliations:** 1 Department of Electrical and Computer Science, Khalifa University, Abu Dhabi, UAE; 2 Department of Biomedical Informatics, School of Medicine and College of Engineering and Applied Sciences, Stony Brook University, Stony Brook, New York, United States of America; 3 Department of Computer Science & Information Systems, University of London, Birkbeck College, London, United Kingdom; 4 Department of Pathology, University of Utah, Salt Lake City, Utah, United States of America; Universitatsmedizin Greifswald, GERMANY

## Abstract

A traceable biomarker is a member of a disease’s molecular pathway. A disease may be associated with several molecular pathways. Each different combination of these molecular pathways, to which detected traceable biomarkers belong, may serve as an indicative of the elicitation of the disease at a different time frame in the future. Based on this notion, we introduce a novel methodology for personalizing an individual’s degree of future susceptibility to a specific disease. We implemented the methodology in a working system called **S**usceptibility **D**egree to a **D**isease **P**redictor (**SDDP**). For a specific disease *d*, let *S* be the set of molecular pathways, to which traceable biomarkers detected from most patients of *d* belong. For the same disease *d*, let *S*′ be the set of molecular pathways, to which traceable biomarkers detected from a certain individual belong. SDDP is able to *infer* the subset *S*′′ ⊆{*S*-*S*′} of undetected molecular pathways for the individual. Thus, SDDP can infer undetected molecular pathways of a disease for an individual based on few molecular pathways detected from the individual. SDDP can also help in inferring the *combination* of molecular pathways in the set {*S*′+*S*′′}, whose traceable biomarkers *collectively* is an indicative of the disease. SDDP is composed of the following four components: information extractor, interrelationship between molecular pathways modeler, logic inferencer, and risk indicator. The information extractor takes advantage of the exponential increase of biomedical literature to automatically extract the common traceable biomarkers for a specific disease. The interrelationship between molecular pathways modeler models the hierarchical interrelationships between the molecular pathways of the traceable biomarkers. The logic inferencer transforms the hierarchical interrelationships between the molecular pathways into rule-based specifications. It employs the specification rules and the inference rules for predicate logic to infer as many as possible undetected molecular pathways of a disease for an individual. The risk indicator outputs a risk indicator value that reflects the individual’s degree of future susceptibility to the disease. We evaluated SDDP by comparing it experimentally with other methods. Results revealed marked improvement.

## Introduction

Biomarkers are biological molecules present in blood, bodily fluids, and tissues. They are classified as either direct or indirect disease markers [1]. Direct biomarkers are directly indicative of a disease. Biomarkers can help in the early detection and diagnosis of diseases. This in turn facilitates the prevention of diseases and promotes potential therapeutic targets. Thus, biomarkers are a good means for determining individuals with subclinical diseases before their progression to clinical diseases [1].

Combining several biomarkers can identify individuals with high risk for developing a disease than individual biomarkers [2,3]. Therefore, compiling biomarker data from multiple literature associated with biomarker-disease associations has become a necessity for maximize the pool of biomarkers. However, compiling such large-scale pool of biomarkers is infeasible due to the practical challenges and resource costs involved [4]. This has led some current computational methods to take advantage of the exponential increase in biomedical literature as a rich source of biomarker information [5]. For example, MEDLINE database [6], which currently indexes more than 2.5 million articles, contains valuable information related to biomarkers. These methods employ text mining techniques for extracting and analysing biomarker-disease association from the literature. However, these methods have not been able to sufficiently coordinate between the findings of the different literature associated with biomarkers. This has resulted in the lack of the following:

- Accuracy of extracting biomarker-disease terms.
- Identifying the common biomarkers that test positive among most patients with a specific disease.
- Knowledge of the combination of biomarkers, whose collective presence is likely to induce a specific disease.

We propose in this paper a novel methodology for personalizing an individual’s degree of future susceptibility to a specific disease. The methodology was developed in such a way that it overcomes the limitations of current methods outlined above. We implemented the methodology in a working system called **S**usceptibility **D**egree to a **D**isease **P**redictor (**SDDP**). The proposed system SDDP is able to predict the degree of future susceptibility to a specific disease for an individual. It is composed of the following four components: information extractor, interrelationship between molecular pathways modeler, logic inferencer, and risk indicator.

The information extractor extracts from biomedical literature the common traceable biomarkers of a specific disease. The component employs novel *strict* rule-based information extraction techniques constructed based on established linguistic theories. These strict rules ensure that *only* the traceable biomarker terms that are closely associated with a disease’s term are extracted.

The interrelationship between molecular pathways modeler models the hierarchical interrelationships between the molecular pathways, to which the traceable biomarkers extracted by the information extractor belong. This helps in inferring the *combination* of molecular pathways, whose traceable biomarker*s collectively* is an indicative of the disease.

The logic inferencer transforms the hierarchical interrelationships between the molecular pathways into rule-based specifications. It also infers all an individual’s undetected molecular pathways of the disease based on a few molecular pathways of the disease, to which traceable biomarkers detected from the individual belong. This is crucial because, the more molecular pathways of a disease inferred for an individual, the more accurate is the prediction of his/her degree of future susceptibility to the disease. With reference to the hierarchical interrelationships between the molecular pathways, the component first composes rule-based specifications that reflects the relationships between the molecular pathways of a specific disease. Then, the component uses the *initial* molecular pathways, whose traceable biomarkers were detected, as given premises to recursively trigger the appropriate specification rules by applying the *standard inference rules* of predicate logic. This leads to inferring as many as possible molecular pathways of the disease for the individual.

Each different combination of molecular pathways, to which detected traceable biomarkers belong, gives a different indication of future degree of susceptibility to the disease [2,3]. SDDP employs this fact to serve as an indicative of future elicitation of the disease for a specific individual. Towards this, the risk indicator component assigns a risk indicator value for the individual’s degree of future susceptibility to the disease based on his/her inferred combination of deficient molecular pathways.

We provide description and limitation of current approaches in Section “Related Work”. We provide our motivation and outline of the approach in Section “Motivation and Outline of the Approach”. We describe the Information Extractor, Interrelationship between MPs Modeller, Logic Inferencer, and Risk Indicator components of SDDP in Sections “Information Extractor”, “Interrelationship between MPs Modeller”, “Logic Inferencer”, and “Risk Indicator”, respectively. We experimentally evaluate the information extraction and ranking features of SDDP in Sections “Evaluating the Information Extraction Feature of SDDP Experimentally” and “Evaluating the Ranking Feature of SDDP Experimentally”, respectively. We provide our conclusion in Section “Conclusion”.

## Related work

### Description of current approaches

Most current computational methods that attempt to identify the risk factors associated with a disease employ statistical-based or text mining-based techniques. Some of these methods investigated single nucleotide polymorphisms (SNPs) genetic variants for their role in diseases. They employed statistical methods (such as logistic regression and neural networks) and several non-parametric techniques (such as the set association technique) [7]. Frau et al. [8] employed network medicine and systems genomics approaches to identify genetic variations associated with diabetes and 12 other traits. The authors could identify a set of 38 genetic variants with cross traits effects. Kycia et al. [9] employed epigenomic and functional genomic approaches to identify the mutated genes associated with a disease. The authors discovered a possible role of C2CD4B and C2CD4A genes as therapeutic targets for preventing diabetes. Vana et al. [10] investigated the characteristic features of mutated genes and their levels of risk associated with a disease. The authors identified protein encoding genes, whose mutations have great impact on the development of diabetic condition.

The relationships among disease-related proteins were investigated in [11] using the SciMiner text-mining tool [12], which uses a dictionary and rule-based technique for recognizing biological terms in texts. The extracted dataset contained 26,716 relationships between disease-related proteins. Einarson et al. [13] extracted data from literature published between 2007 and 2017 to estimate the prevalence of CVD among patients with diabetes. The results showed that CVD is a major cause of comorbidity among patients with diabetes. Abbasi et al. [14] extracted relationships between diseases’ incidents and 167 blood and urinary-based markers.

A number of studies employed logic-based computational methods to infer the risk factors associated with a disease. Wynn et al. [15] demonstrated that logic-based models can be used effectively to perform biological inferences about the fundamental characteristics of molecular networks. Jafari et al. [16] demonstrated that logic-based methods are useful for improving static conceptual models in molecular biology. Palù et al. [17] demonstrated that logic-based models can be used effectively for predicting protein structures and functions.

Inflammatory markers play a role in the progression and development of diseases [18]. For example, about 127–129 inflammatory cytokines have been elevated on the onset of a disease [18]. Several methods employed data mining techniques for investigating these markers as potential predictors of the development of diseases. Some of these methods found elevated levels of markers among individuals with diseases [19].

### Limitations of the current approaches

We outline below the four major limitations of the current approaches described in Subsection “Description of Current Approaches”:

1. *Assessing only a single outcome*: Often, traditional biomarker methods assess only a single (or a few) biomarker for its association with a disease [20]. Investigating only a single biomarker is likely to have inherent limitations. Most of these methods target only specific case studies or/and risk factors. However, most diseases are multifactorial where several biomarkers and risk factors are involved. Moreover, these methods become unstable as more SNPs are identified [7]. This is because, as the number of parameters surpasses the number of cases, parameter fluctuation estimates become extremely large.
2. *Inability to compose a clear disease’s combination of biomarkers*, *whose collective presence is an indicative of the disease*: Each *different combination* of deficient molecular pathways of a disease gives a different indication of the susceptibility to a disease [2,3]. Current methodologies have not come up with a clear disease’s combination of biomarkers, whose collective presence is likely to induce the disease. This is because, in part, identifying a combination of biomarkers for each disease requires a large number of phenomic associations to be at hand, which in turn, requires a large number of biomarkers to be confirmed by many clinical outcomes [2].
3. *Performing predictions that may not always be accurate*: The more molecular pathways of a disease detected for an individual, the more accurate is the prediction of his/her degree of future susceptibility to the disease. However, most current methodologies base their predictions of a disease on *only a few* deficient molecular pathways, whose biomarkers test positive. This is because the procedure requires a large number of biomarkers to be checked beforehand by medical tests, which is laborious and expensive. Therefore, the predictions of these methodologies may not always be accurately.
4. *Associating unrelated biomarker-disease terms*: Most of the information extraction methods in the area of biomarkers employ NLP-based techniques that do not follow *strict* linguistic principles. This results in associating too many biomarker-disease terms, some of which are not really associated. Employing *strict* NLP techniques is crucial, especially when applied to large literature that lack standardization, which is the case in current biomedical literature. Moreover, most of the methods that employ statistical techniques (such as logistic regression and neural networks) may produce many false positive results, if the number of predictor variables becomes rather large [21].

## Motivation and outline of the approach

### Motivation

To the best of our knowledge, this is the first research work that combines the following three techniques for predicting an individual’s degree of future susceptibility to a specific disease: information extraction, inference rules of predict logic [22], and modeling the interrelationships among the molecular pathways of a specific disease. We implemented each of the three techniques in a separate component of the SDDP system. Combining the three techniques (i.e., components) enables SDDP to overcome the limitations of current methods outlined in Subsection “Limitations of the Current Approaches”. Moreover, this is the first research work, to the best of our knowledge, that employs the inference rules of predict logic [22] to infer as many as possible undetected molecular pathways of a disease for an individual based on a few molecular pathways of the disease, to which traceable biomarkers detected from the individual belong. The logic-based inference component of SDDP ensures that the *collective combination* of inferred molecular pathways of a disease for an individual, whose traceable biomarker*s* were detected from the individual, is likely be an indicative of the disease. Combining the three techniques enables SDDP to overcome the four major limitations of the current approaches outlined previously as follows:

- *Overcoming limitation 1*: SDDP overcomes this limitation by assessing the association between any number of detected traceable biomarkers. Actually, the more traceable biomarkers of a disease detected for an individual, the more accurate is the SDDP’s prediction of the individual’s degree of future susceptibility to a disease. SDDP extracts biomarker data from a large number of study sources to quantify biomarker-disease associations. This facilitates subgroup analyses, which leads to more accurate biomarker exposure estimation [2,3]. This also enables SDDP to investigate with better accuracy the following: (1) a large biomarker exposure range, and (2) biomarker population subgroups and their associations with specific diseases.
- *Overcoming limitation 2*: SDDP overcomes this limitation by composing a clear list of the common traceable biomarkers that is detected in most patients with a specific disease. It could so by coordinating and integrating the findings/data found within 463,331 biomedical literature that focus on biomarkers. The outcomes of current methods in coordinating between the findings/data of these studies have not been sufficient enough to compose clear lists of the common traceable biomarkers detected in most patients with a specific disease. Also, SDDP overcomes limitation 2 by predicting the *smallest and tightly defined set* of molecular pathways that elicit a specific disease. It does so by modeling the hierarchical interrelationships between the molecular pathways of a specific disease based on their overlapping characteristics.
- *Overcoming limitation 3*: SDDP overcomes this limitation by personalizing an individual’s degree of future susceptibility to a specific disease. It does so by employing the inference rules of predict logic to infer as many as possible undetected molecular pathways of a disease for an individual based on a few molecular pathways of the disease, to which traceable biomarkers detected from the individual belong.
- *Overcoming limitation 4*: SDDP overcomes this limitation by employing novel *strict* NLP rule-based information extraction techniques. These strict rules enable SDDP to extract *only* the traceable biomarkers terms that are closely associated with a disease terms based on the structure of sentencese. SDDP extracts only traceable biomarker-disease pair of terms that co-occur: (a) significantly within texts, and (b) in significant number of texts. Towards this, SDDP computes terms’ co-occurrence probabilities using Z-score [23], where two terms are considered to be associated, if their co-occurrence probability of being associated is significantly greater than being unassociated.

### Outline of the approach

**Fig 1:**
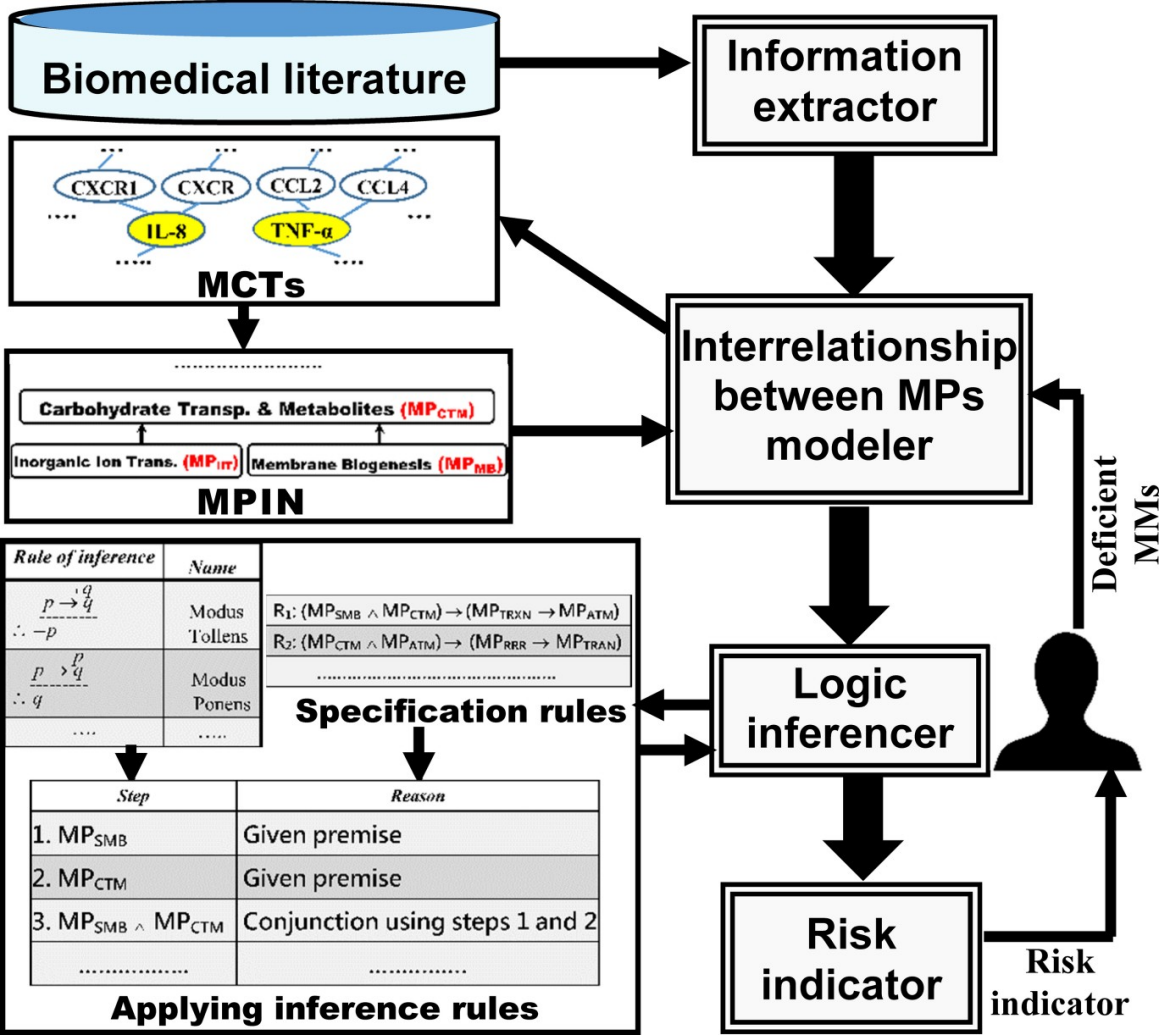
SDDP system architecture.

Fig 1 presents the system architecture. It shows the relationships between the four components comprising our proposed system SDDP. Table 1 presents abbreviations of key terms used in the paper. We define below key concepts used in the paper:

- *Disease molecular pathway*: It is a pathway that is known to have at least one disrupted molecule associated with a disease. For example, estrogen receptor, overexpression, and EGFR are pathways for breast, gastric, and colorectal cancers, respectively. The involved molecules share specific recognizable phenotypic pattern and interacting signaling pathways, which can be manifested in the disease regulatory molecular network. This is caused by deregulation of the *molecular* network of the disease, which can result in disordered physiological processes associated with the disease. Such data can be obtained from clinical notes about patients’ diseases.
- *Molecular Characteristic Tree*: It is a representational model that depicts the hierarchical interrelationships between the molecules associated with a specific disease based on their overlapping biological characteristics.
- *Molecular Pathway Interrelationships Network*: It is a network representing the hierarchical interrelationships between the molecular pathways of a disease based on their shared molecules manifested in the disease’s Molecular Characteristic Trees.
- *Biomarker*: It is a measurable substance, process, or structure indicator of a disease. In this work, we only consider measurable molecular substances (i.e., traceable biomarkers) that predict a patient’s specific disease.

**Table 1:** Abbreviations of key terms used in the paper.

*Term*	*Abb*.	*Term*	*Abb*.
Molecule Pathway	**MP**	Natural Language Processing	**NLP**
Molecular Marker	**MM**	MP Interrelationships Network	**MPIN**
Type 2 Diabetes	**T2D**	Molecular Characteristic Tree	**MCT**
Part Of Sentence Tree	**POST**	Single Nucleotide Polymorphism	**SNP**

With reference to the system architecture in Fig 1, we outline below the sequential processing steps taken by SDDP to predict the degree of future susceptibility to a specific disease for an individual:

1. ***Information extractor component*:** This component extracts from biomedical literature the common MMs that test positive among most patients with a specific disease. Section “Information Extractor” describes this process in details.
2. ***Interrelationship between MPs modeller component*:** This component models the hierarchical interrelationships between the molecular pathways of a specific disease, whose MMs were extracted by the information extractor component. The component performs the modelling through the following steps:
  - ***Constructing MCTs*:** The component constructs Molecular Characteristic Trees (MCTs) for each set *S* of MMs that belongs to a same molecular pathway. Each tree is rooted at one of the MMs in the set *S*. Section “Constructing MCTs” describes this process in details.
  - ***Constructing MPIN*:** The component constructs a MP Interrelationships Network (MPIN) representing the hierarchical interrelationships between the MPs of the disease based on their shared biological characteristics manifested in their MCTs. Section “Constructing MPIN” describes this process.
3. ***Logic inferencer component*:** This component applies the inference rules for predicate logic to infer as many as possible undetected molecular pathways of a disease for an individual based on a few molecular pathways of the disease, to which MMs detected from the individual belong. The component performs the inferencing through the following two steps:
  - ***Composing rule-based specifications*:** The component composes specification rules that reflect the interrelationships between the different MPs of a disease. It composes these rules with reference to the MPIN (recall step 2-b). Section “Composing Rule-Based Specifications” describes this process in details.
  - ***Applying the inference rules for predicate logic*:** This component uses the initial molecular pathways, whose biomarker molecules tested positive by medical screening for the individual, as given premises to recursively trigger the appropriate specification rules. It does so by applying the *standard inference rules* for predicate logic. Section “Applying the Inference Rules for Predicate Logic” describes this process.
4. ***Risk indicator component*:** Based on the combination of molecular pathways of a disease inferred by the logic inferencer component for an individual, this component outputs a risk indicator value. The indicator reflects the individual’s degree of future susceptibility to the disease.

## Information extractor

For most diseases, there are currently unclear lists of the *common* MMs that test positive among most patients with these diseases [4]. However, there has been an exponential increase in biomedical literature associated each disease, which can be used as sources for composing these lists. Unfortunately, there is a lack of coordination between the data found within these different studies. Moreover, there is a lack of coordination between the findings of these studies [24]. This has significantly diminished the effectiveness of these studies. SDDP takes advantage of this literature to computationally and automatically extract from them the common MMs that test positive among most patients with a specific disease. SDDP will employ the extracted MMs to model the hierarchical interrelationships between the MPs containing these MMs.

We first retrieve the biomedical literature associated with a specific disease from a reputable biological database. In the implementation of SDDP, we retrieved the abstracts of biomedical literature from PubMed [25]. We process the abstracted biomedical literature using the Java library of OpenNLP [26]. OpenNLP provides the following services for processing Natural Language texts. It parses, tokenizes, segments sentences, tags Part-Of-Speech (POS), recognizes and extracts named entity, and provides co-reference resolution, etc. From each set of publications associated with a specific disease, SDDP extracts the MM terms that are semantically related to the disease terms. We retrieved human genes, genetic disorders, and traits from the Online Mendelian Inheritance in Man (OMIM) [27,28]. We retrieved expression profiles of human protein coding genes from the Human Protein Atlas (HPA) [29].

The co-occurrence of a molecular term *t* and a disease term *d* in a sentence may not always be an indicative that *t* and *d* are really related [30]. For *t* and *d* to be associated, they have to be semantically related in the sentences. SDDP employs novel computational linguistic techniques for extracting the MM terms that are semantically related to a disease term. The techniques consider not only the explicit co-occurrences of terms but also their implicit co-occurrences in sentences.

We composed novel strict information extraction NLP-based rules that govern the extraction of semantically related terms. The rules are constructed based on established linguistic principles. We investigated many linguistics principles to compose the strict rule-based techniques. These strict rules ensure only the MM terms that are semantically related to a disease terms are extracted based on the structure of sentences. These rules overcome the limitations of most current NLP-based information extraction techniques, which may associate terms that are not necessarily related.

In the framework of SDDP, a sentence is perceived as a hierarchical structure comprised of constituents (i.e., clauses, units, and groups) [31]. Constituents are determined based on a set of linguistic rules that define the Constituents’ appropriate syntactic composition. Each sentence is conceived as a ordered rooted tree called POST. The tree reflects the syntactic structure of a sentence based on its constituents’ hierarchical dependency relations. SDDP considers a MM term and a disease term to be semantically related, if their co-occurrence probability of being semantically related is significantly greater than being unrelated in sentences. Towards this, it computes the co-occurrence probabilities of MM-disease terms using Z-score [23].

Due to space limitation, we describe below only two of our proposed strict NLP-based information extraction rules. The first rule is based on the linguistic principle that states an antecedent is closely related to subsequent terms in sentence, if these terms are linked to the antecedent by some pronoun (e.g., “whom”, “which”, “it”, “who”, and “that”) [32]. Based on this, we replace each pronoun with the *closest* terms that are part of the predecessor independent clause. The second rule is based on the linguistic principle that states a pair of independent clauses linked by a preposition modifier (e.g., “whereas”, “while”, and “but”) is unrelated [33,34]. Based on this, we consider the co-occurrence of a MM-disease pair of terms *unrelated*, if each of the terms is mentioned in a separate independent clause and the two independent clauses are linked by a preposition modifier. We present the complete set of our proposed NLP rules in Appendix A of the supplemental material in S1 File.

**Fig 2:**
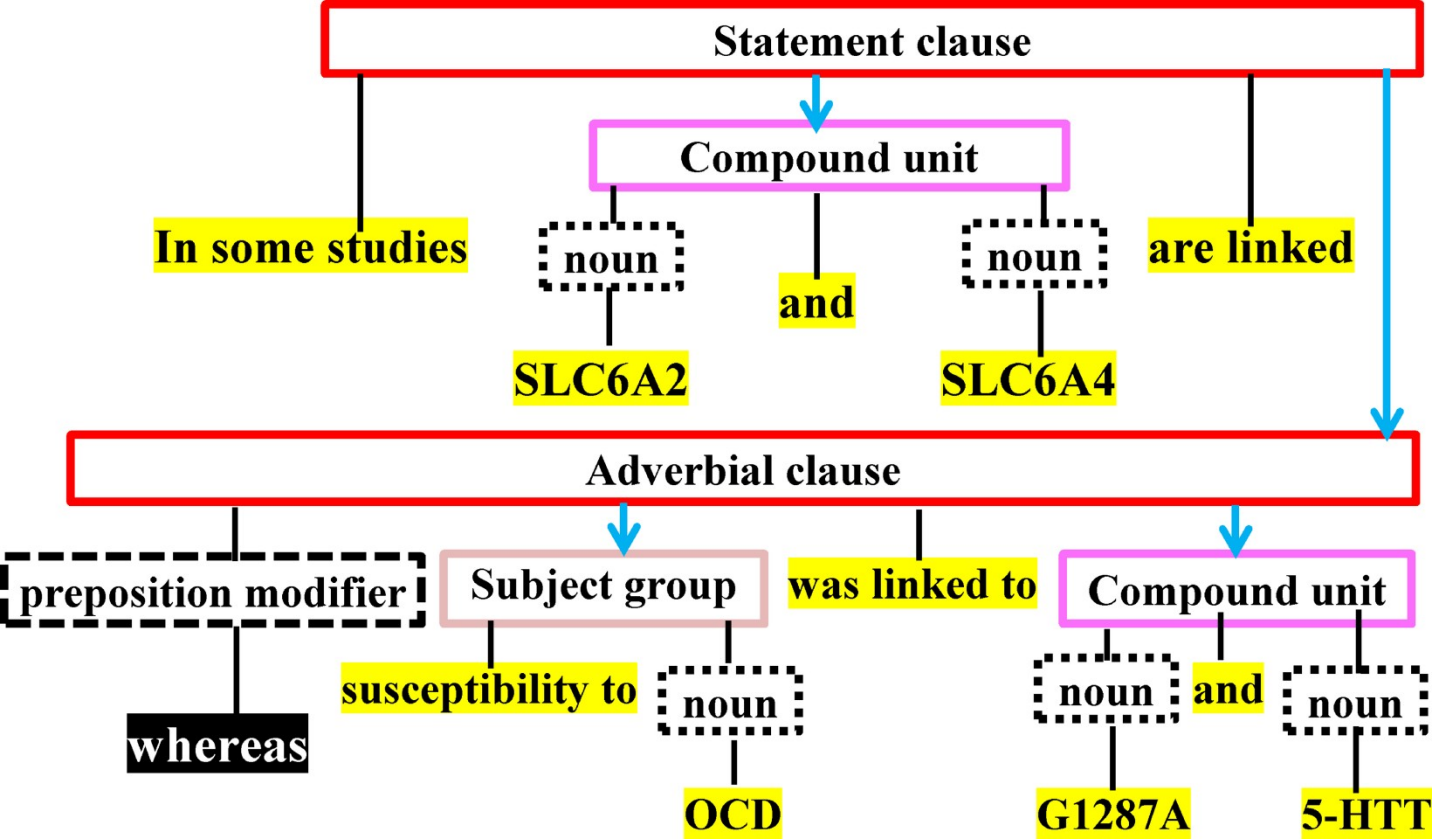
POST for the sentences presented in Example 1. A compound unit consists of two or more conjuncts connected by one or more coordinators. A conjoint clause is a constituent linked to another constituent by a coordination. An adverbial clause contains conjunctive adverbs.

**Example 1**. Consider Fig 2 and the following sentence: “In some studies, SLC6A2 and SLC6A4 are linked, whereas susceptibility to OCD was linked to G1287A and 5-HIT”. Each of the nouns “SLC6A4” and “SLC6A2” is unrelated to each of the nouns “5-HIT”, “OCD”, and “G1287A”, because the two sets of nouns are connected by the preposition modifier “whereas”. The nouns “SLC6A4” and “SLC6A2” are related. The nouns “5-HIT”, “OCD”, and “G1287A” are related.

## Interrelationship between MPs modeller

### Constructing MCTs

Most molecules associated with a disease have overlapping biological characteristics. To account for these shared characteristics, we construct Molecular Characteristic Trees (MCTs) for each MP of a specific disease. An MCT models the hierarchical interrelationships between the molecules of a MP based on their overlapping biological characteristics. A set of MCTs are constructed for each MP. The number of these MCTs is the number of the MMs extracted by the information extractor component that belongs to the MP. Each MCT will be rooted at a node representing one of the MMs of the MP. Let *S* be a set of MPs of a specific disease, whose MMs were extracted by the information extractor component. To account for the common biological characteristics among the molecules of each MP *MP* ∈*S*, we construct MCTs for *MP*. Each MCT *mct* that belongs to *MP* is constructed as follows. *mct* will be rooted at a node *n*_*i*_ representing a MM *mm*∈*MP*. Each molecule *mol* that is biologically related to *mm* is represented by a node *n*_*j*_ and is connected to *n*_*i*_ by an edge. The molecules that are biologically related to *mol* are represented by nodes, which will be connected to *n*_*j*_.

That is, an MCT is the outcome of transforming a molecular network structure into a hierarchical tree-like structure for the purpose of identifying the relative associations of molecules to a root molecule node. The transformation from a network structure to a tree structure is performed by removing multiple parentage of nodes. That is, each node should have only one parent node. This is accomplished by performing bottom-up search runs for each branch structure starting from the root node. At each run, all associations (i.e., edges) that connect a node *n* to the root node are removed except for the one that maintains the shortest path from *n* to the root. If there are more than one shortest path from *n* to the root, only one of them is selected at random.

**Fig 3:**
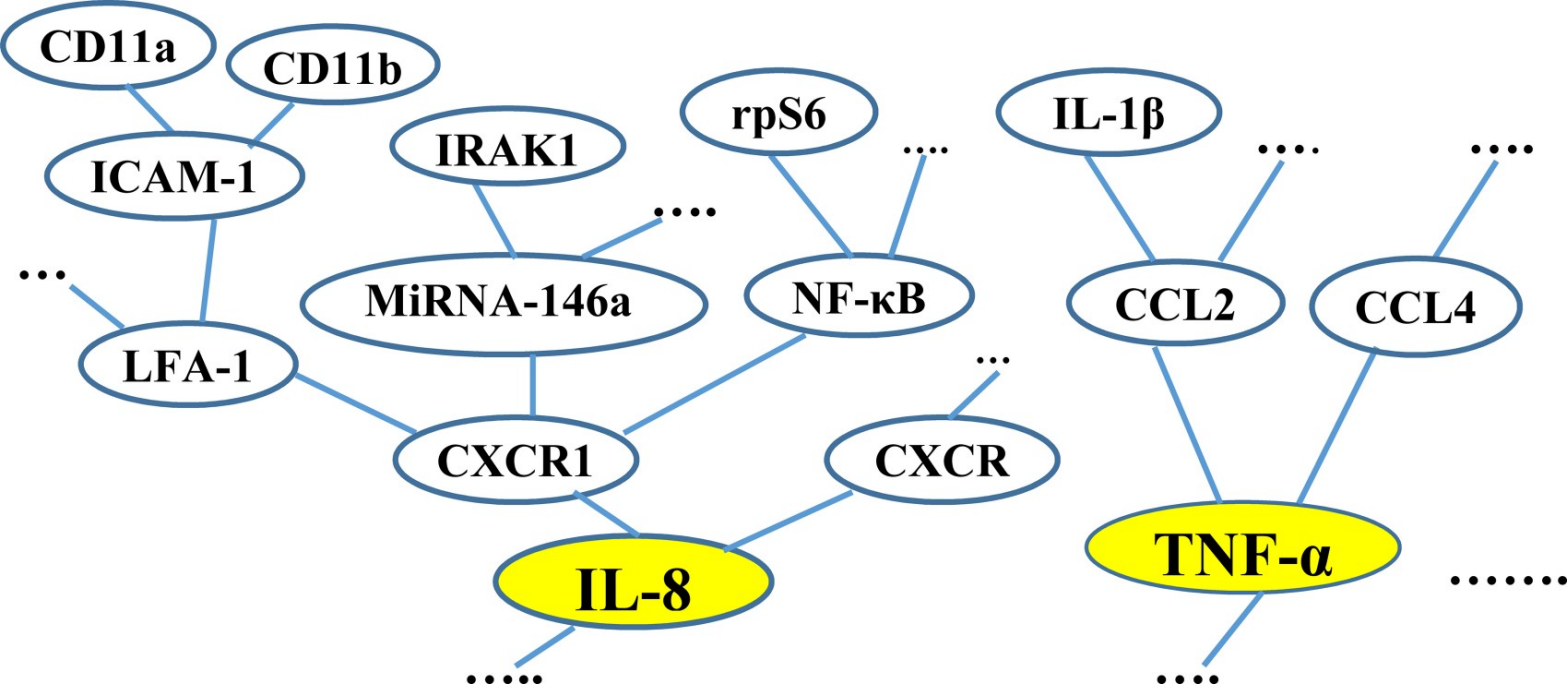
A fragment of the MCTs for the MP “CXC chemokine” associated with T2D.

**Example 2 (running example)**. Consider the MP “CXC chemokine”, which is involved in T2D. Fig 3 shows a fragment of the MCTs for “CXC chemokine”. The two MCTs shown in the figure are rooted at the MMs “IL-8” and “TNF- α”, which belong to “CXC chemokine” and are involved in T2D. The figure shows fragments of the interrelationships between some of the molecules related to the two MMs.

### Constructing MPIN

To infer the MPs, to which detected traceable biomarkers belong, we need to identify their interrelationships. These interrelationships will be transformed by SDDP into inference specification rules, which will be used by the system to infer as many as possible undetected molecular pathways of the disease for an individual. Towards this, we construct a network representing the hierarchical interrelationships between the MPs of the disease based on their shared molecules manifested in the MCTs of these MPs. We call the resulting network **MP I**nterrelationships **N**etwork (**MPIN**).

An MPIN is constructed as follows. Each set of molecules that belongs to a MP *MP*_*x*_ is represented by a node named *MP*_*x*_ in the MPIN. Two MPs *MP*_*x*_ and *MP*_*y*_ in the MPIN are linked by an edge, if there is at least on common molecule shared by *MP*_*x*_ and *MP*_*y*_. That is, *S*(*MP*_*x*_) ∩ *S*(*MP*_*y*_) ≠ ∅, where *S*(*MP*_*x*_) and *S*(*MP*_*y*_) are the sets of molecules that belong to *MP*_*x*_ and *MP*_*y*_, respectively. The hierarchical relationship between *MP*_*x*_ and *MP*_*y*_ is depicted in the MPIN based on the hierarchical level of their *lowest common molecule* relative to: (1) the lowest root node among the set of root nodes of the MCTs that belong to *MP*_*x*_, and (2) the lowest root node among the set of root nodes of the MCTs that belong to *MP*_*y*_.

Let *n*_*com*_ be the lowest common molecule node. Let *n*_*x*_ be the lowest molecule root node of *MP*_*x*_. Let *n*_*y*_ be the lowest molecule root node of *MP*_*y*_. Let $\bar{x}$ be the hierarchical level of *n*_*com*_ with regard to *n*_*x*_. Let $\bar{y}$ be the hierarchical level of *n*_*com*_ with regard to *n*_*y*_. If $\bar{x}$ < $\bar{y}$, an arrow originating from *MP*_*y*_ to *MP*_*x*_ is initiated in the MPIN to depict the hierarchical relationship between *MP*_*y*_ and *MP*_*x*_. In this case, *MP*_*x*_ is more specific than *MP*_*y*_.

**Fig 4:**
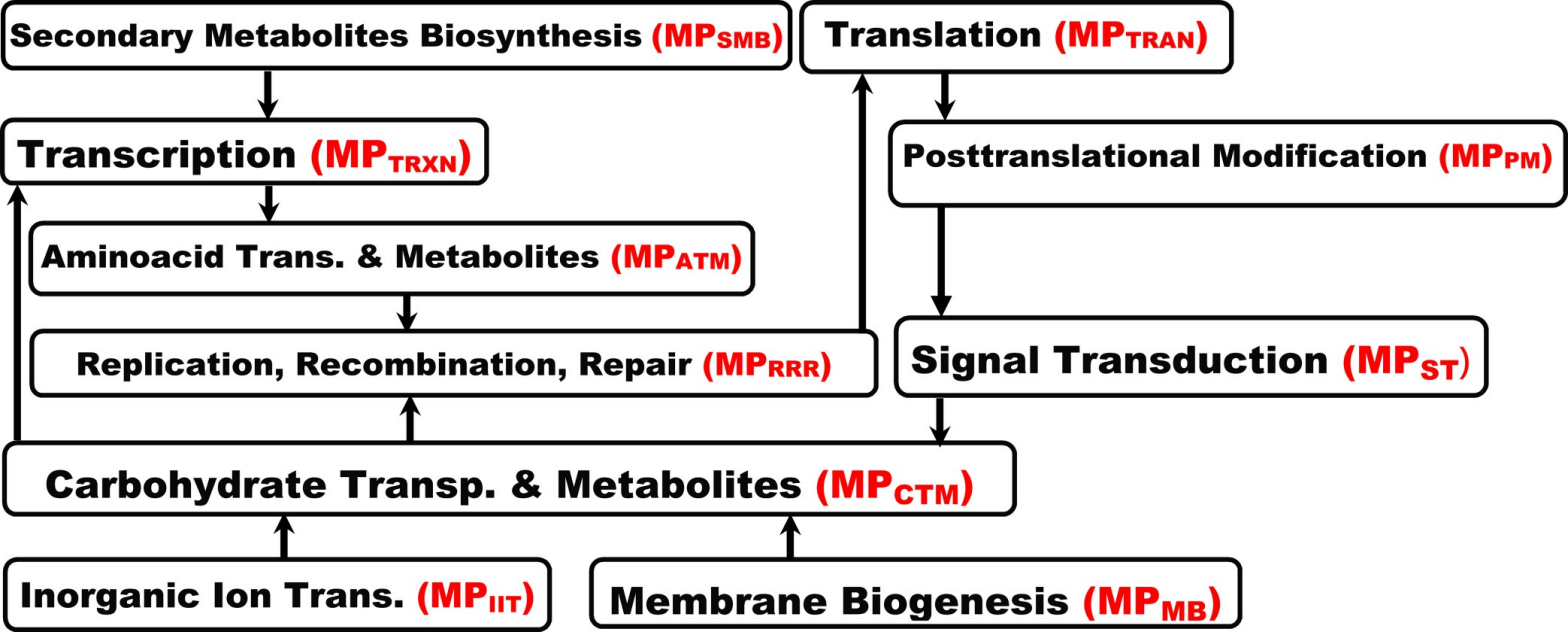
A fragment of MPIN depicting the hierarchical interrelationships between MPs associated with T2D in our running example. MP_xyz_ denotes the MP, whose name abbreviation is xyz.

**Example 3 (running example)**. Fig 4 shows a fragment of MPIN depicting the interrelationships between the MPs associated with T2D in our running example.

## Logic inferencer

### Composing rule-based specifications

We compose rule-based specifications that reflect the interrelationships between MPs, whose detected traceable biomarker*s* collectively is an indicative of a disease. Eventually, these rules will be used by SDDP as inference rules to infer as many as possible undetected molecular pathways of a disease for an individual based on a few molecular pathways, to which traceable biomarkers detected from the individual belong.

We composed these rules with reference to the MPIN that depicts the interrelationships between MPs. Towards this, we convert the interrelationships between the MPs manifested in the MPIN into transformation rules. Specifically, we convert the hierarchical interrelationships between the MPs in the MPIN by chaining them together into logical transformation rules.

We compose the rule-based specifications in a format resemble the premises of predicate logic [23]. A predicate is a logical statement composed of one or more variables. It is transformed to a proposition by connecting its statements by logical connectives. In the framework of SDDP, the specification rules are developed in the same manner. Specification rules are updated periodically to reflect newly discovered MMs for a disease or/and newly published works about the disease.

**Fig 5:**
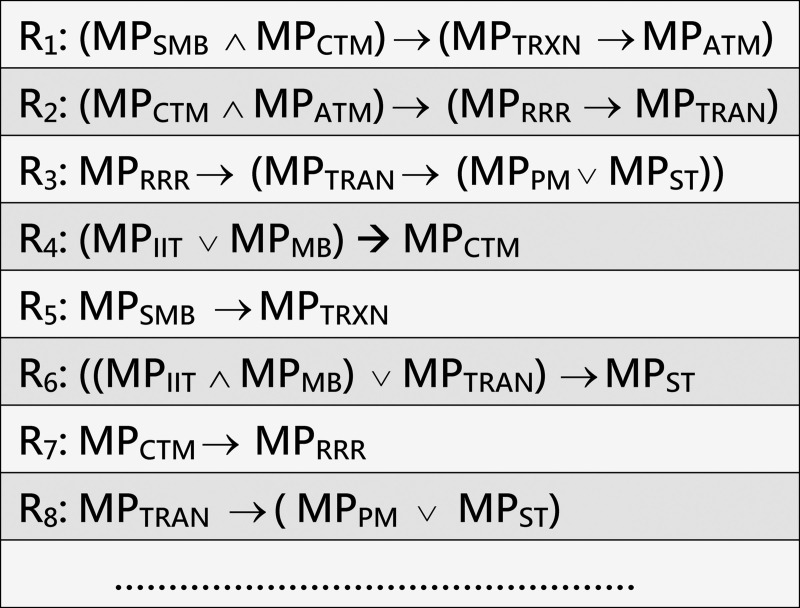
A sample of specification rules that reflect the interrelationships between MPs associated with T2D constructed with reference to the MPIN in Fig 4. R_i_ denotes rule/premise number *i*. The logic symbols “∧”, “∨”, and “→” denote conjunction, logical disjunction, and implies respectively.

**Example 4 (running example)**. Fig 5 shows a fragment of specification rules that reflect the interrelationships between the MPs associated with T2D. They are constructed with reference to the MPIN in our running example in Fig 4. A complete list of these rules is shown in Appendix B of the supplemental material in S1 File.

### Applying the inference rules for predicate logic

The more molecular pathways of a disease inferred for an individual, the more accurate is the prediction of the individual’s degree of future susceptibility to the disease. Therefore, we propose to use the inference rules of predict logic [22] to infer as many as possible undetected molecular pathways of a disease for an individual based on a few molecular pathways, to which traceable biomarkers detected for the individual belong. In the framework of SDDP, Prolog is used as the logic programming language. This is because Prolog enables us to easily avoid infinite loops by setting the search algorithm for matching predicates to breadth-first and by iterative-deepening using meta-interpreters.

**Fig 6:**
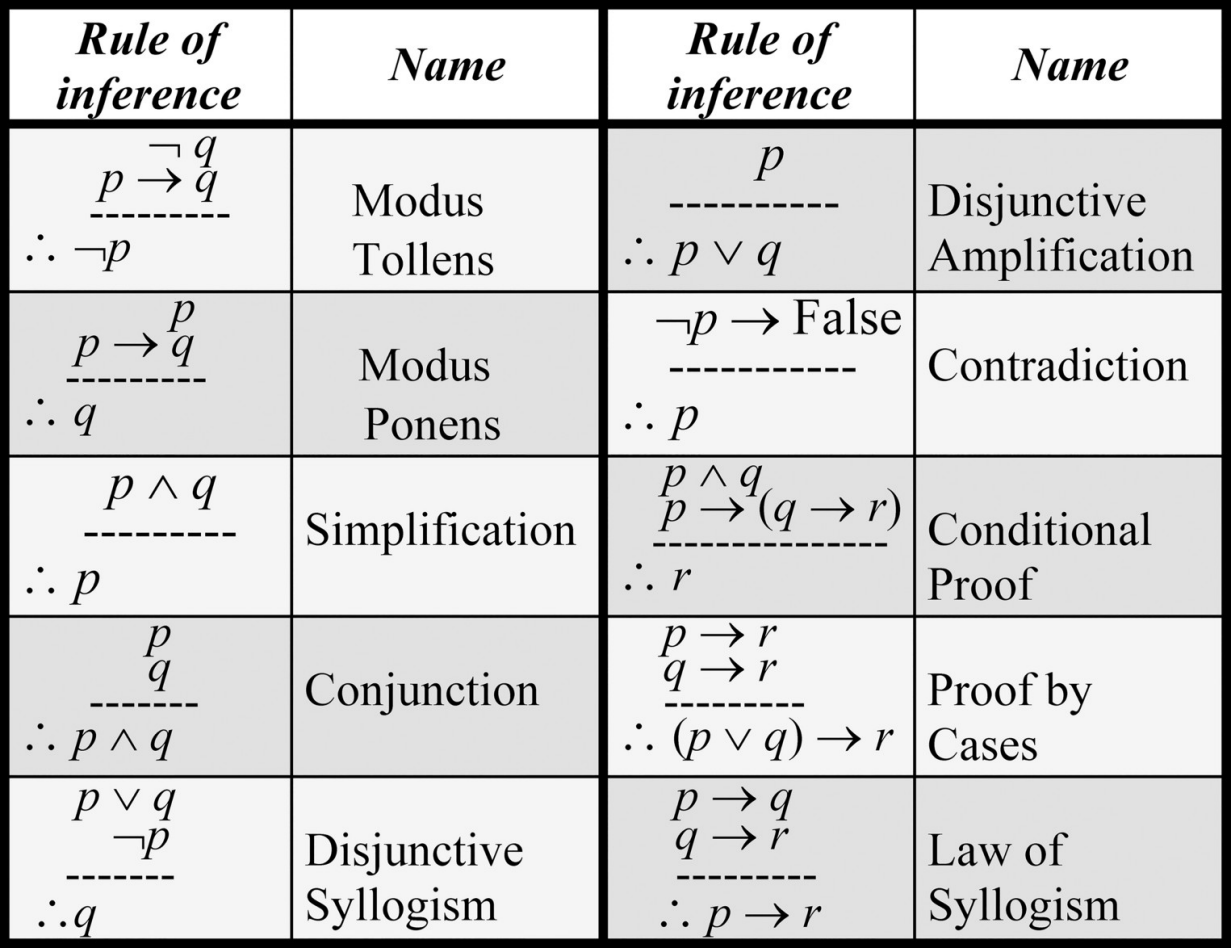
Major standard inference rules for predicate logic.

By matching an individual’s detected traceable biomarkers (e.g., MMs) that revealed abnormalities for a specific disease (e.g., by medical screening) with the corresponding ones in the MCTs of the disease’s MPs, SDDP can identify the *initial* MPs associated with the disease for the individual. SDDP will use these *initial* MPs as given premises to trigger the appropriate specification rules (Section “Composing Rule-Based Specifications”) by applying the *standard inference rules* for predicate logic. This will lead to implicitly infer as many as possible MPs of the disease for the individual. Fig 6 shows the major standard inference rules for predicate logic [23]. Thus, SDDP employs the following for inferring the MPs of a disease for an individual that are indicative of the disease: (1) the specification rules (i.e., premises) of a disease, (2) the initial deficient MPs *(i*.*e*., *given premises)* for an individual identified by medical screening, and (3) the standard inference rules for predicate logic (recall Fig 6).

The specification rules are triggered by applying the standard inference rules for predicate logic. SDDP triggers recursively the specification rules using the given premises, auxiliary inferred premises, and the standard inference rules for predicate logic. At each recursion, a specification rule (i.e., a premise) is triggered and applied to the premises that have been proven previously. This will lead to a newly proven premise. The conclusions will be a set of inferred MPs. The conclusions are valid, if they have been deducted from all previous premises [23].

**Fig 7:**
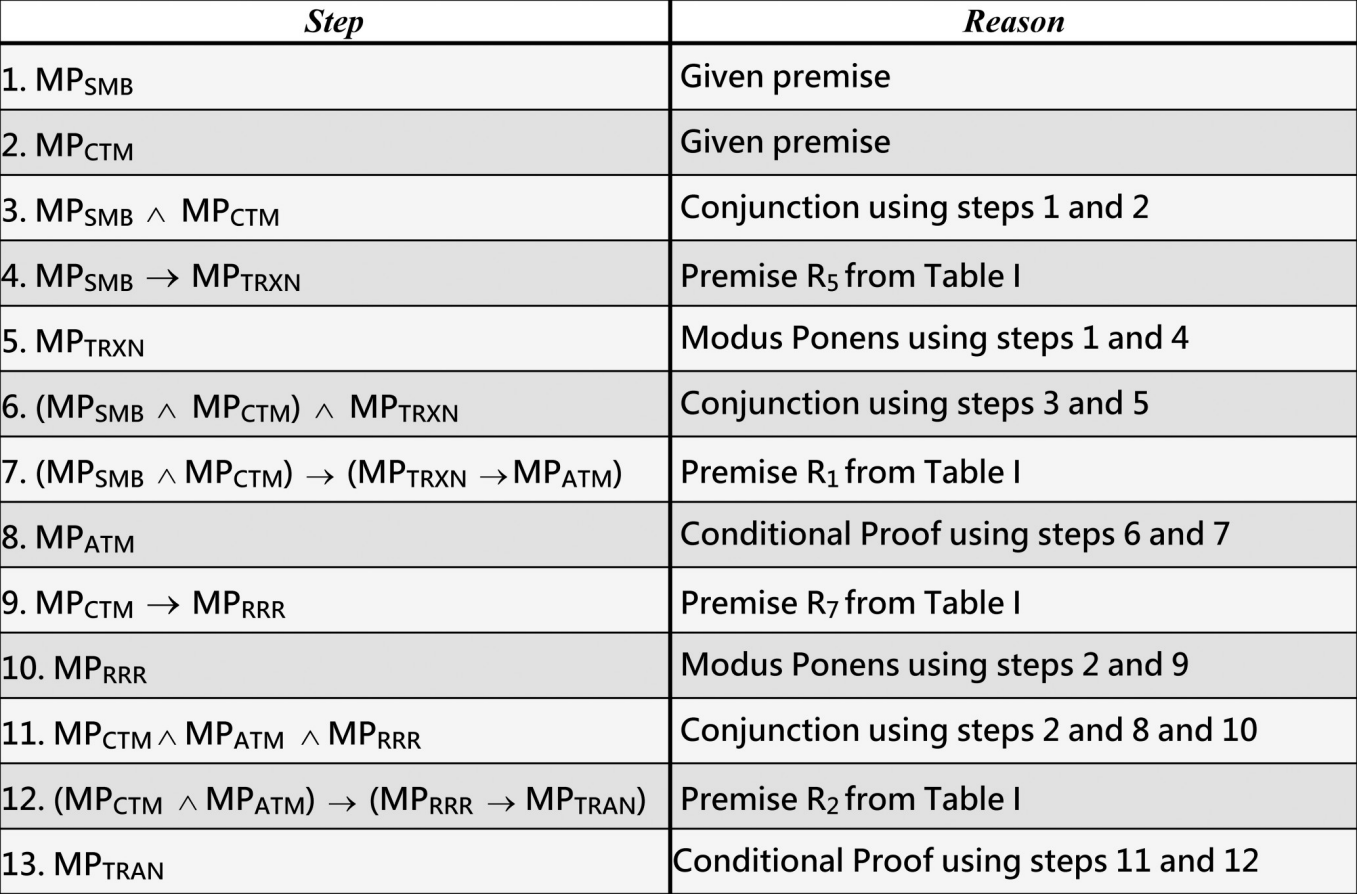
Inferring MP_TRXN_, MP_ATM_, MP_RRR_, and MP_TRAN_ from the given premises MP_SMB_ and MP_CTM_, which are associated with T2D, as described in our running example 5.

**Example 5 (running example)**. Consider that the initial deficient MPs of T2D identified by medical screening for an individual are MPs_MB_ and MP_CTM_. As Fig 7 shows, the inference rules could infer the following four MPs for the individual: MP_TRXN_ (from step 5), MP_ATM_ (from step 8), MP_RRR_ (from step 10), and MP_TRAN_ (from step 13).

## Risk indicator

Each different combination of detected traceable biomarkers’ molecular pathways outputs by component Logic Inferencer gives a different indication of future degree of susceptibility to the disease [2,3]. That is, each different combination of molecular pathways, to which detected traceable biomarkers belong, may serve as an indicative of the elicitation of the disease at a different time frame in the future. Thus, a combination of inferred MPs of a disease for an individual can be an indicative of the individual’s degree of future susceptibility to the disease. This led us to assign a risk indicator value for each combination of identified MPs of a specific disease for a specific individual. Each indicator reflects an individual’s degree of future susceptibility to a disease.

An indicator value is assigned to a combination of MPs of a disease for an individual as follows. Let *S* be the set of MPs of a specific disease. We assign a score to each combination *c* ⊆*S*. The score reflects the degree of association between the combination *c* and the disease. Specifically, it reflects the ***dominance*** status of *c* relative to each other combination *c*′ ⊆ *S*. First, we compute the pairwise *beats* and *looses* for each combination. This is performed based on the co-occurrences of the combination’s MPs in the abstracts of biomedical publications associated with a disease *d* under consideration. Combination *c*_*i*_ beats combination *c*_*j*_, if the number of times that the co-occurrence weight of *c*_*i*_ is greater than that of *c*_*j*_ in abstracts. Eventually, each combination *c* is assigned a score, which is the difference between the number of times that *c* beats the other combinations and the number of times it loses. Finally, the combinations are ranked based on their dominance scores.

An individual is given a risk indicator value that reflects his/her future degree of susceptibility to the disease *d* as follows. Let *c*_*x*_ be the combination of MPs output by component Logic Inferencer for the individual (recall Section “Logic Inferencer”). The individual will be assigned a risk indicator value corresponds to the dominance rank of combination *c*_*x*_. That is, after all MP combinations are ranked based on their dominance scores, the individual will be assigned a risk indicator value corresponds to the dominance rank of *c*_*x*_. In Appendix C of the supplemental material in S1 File, we describe in details how risk indicators are computed.

**Example 6**. Consider that there are ten combinations of MPs: c_1_-c_10_. Consider that the number of co-occurrences of each of the ten combinations in three biomedical publications (p_1_-p_3_) associated with the disease under consideration is as shown in Table 2. Table 3 shows how the score *S*_*t*_ of each of the ten combinations is computed based on its number of occurrences in the three publications presented in Table 2. For example, let c_9_ be the combination of the MPs, to which detected traceable biomarkers from an individual belong. The individual will be given the risk indicator 3 (see the last row in Table 3).

**Table 2:** The number of co-occurrences of each of the ten MP combinations in three publications associated with a disease as described in Example 6.

	c_1_	c_2_	c_3_	c_4_	c_5_	c_6_	c_7_	c_8_	c_9_	c_10_
**p**_**1**_	3	0	0	0	7	0	3	6	3	0
**p**_**2**_	0	3	7	3	0	0	0	3	0	4
**p**_**3**_	3	3	5	0	0	6	0	0	4	0

**Table 3:** Beats/looses scores of the combinations of the MPs described in Example 6 based on their number of co-occurrences on the three publications as shown in Table 2.

	c_1_	c_2_	c_3_	c_4_	c_5_	c_6_	c_7_	c_8_	c_9_	c_10_
**c**_**1**_	0	0	+	-	0	0	-	+	+	-
**c**_**2**_	0	0	+	-	-	0	-	0	+	0
**c**_**3**_	-	-	0	-	-	0	-	-	-	-
**c**_**4**_	+	+	+	0	0	0	0	+	+	+
**c**_**5**_	0	+	+	0	0	0	-	0	0	0
**c**_**6**_	0	0	0	0	0	0	0	+	0	0
**c**_**7**_	+	+	+	0	+	0	0	+	+	0
**c**_**8**_	-	0	+	-	0	-	-	0	-	0
**c**_**9**_	-	-	+	-	0	0	-	+	0	-
**c**_**10**_	+	0	+	-	0	0	0	0	+	0
$S_{c_{i}}$	0	**+1**	**+8**	**-6**	-1	-1	**-6**	**+4**	**+3**	**-2**
**Risk Indicator**	**5**	**4**	**1**	**9**	**6**	**6**	**9**	**2**	**3**	**8**

“+” denotes: combination c_i_ beat combination c_j_. “-” denotes: combination c_i_ lost to combination c_j_. “0” denotes: c_i_ and c_j_ have the same number of beats and looses. *S*_*ci*_ is the dominance score of c_i_.

## Evaluating the information extractor component of SDDP experimentally

We implemented SDDP in Java and ran it under Windows 10 Pro and Intel(R) Core(TM) i7-6820HQ processor. The RAM and CPU of the machine have 16 GB and 2.70 GHz respectively. The objective of this test is to evaluate the quality of the information extraction feature of SDDP. That is, we aim at evaluating the impact of the information extractor component of SDDP on its prediction accuracy. This is because we wanted to evaluate our novel linguistic *strict* rule-based information extraction techniques employed by the information extractor component. As described in Section “Information Extractor”, these strict rules were constructed to ensure that *only* the biomarker terms (e.g., mutated genes biomarkers) that are closely associated with a disease are extracted.

- We evaluated the information extraction feature of SDDP by comparing it with the following four text mining methodologies: SCAIView [24,35], AEGDA [36], BeFree [37], and PKDE4J [38]. For the evaluations, we used the ***reported results*** of AEGDA, BeFree, and PKDE4J. Specifically, we used the reported results of AEGDA, BeFree, and PKDE4J in [36]. Moreover, we used the ***same four gold stand corpora and setting*** described in [36]. As for the evaluation of SCAIView, we performed the following: (1) used the latest version of SCAIView Academia [35] (accessed date May 31, 2020), and (2) ran queries consisting of a MeSH [39] disease name and the keyword human genes. We evaluated SDDP using the same 10-fold cross-validation strategy reported in [36]. Below are brief descriptions of SCAIView, AEGDA, BeFree, and PKDE4J:
- ***SCAIView [35,24]***: SCAIView incorporates the following two external software components for retrieving biomedical literatures: ProMiner (named entity recognition tool) and SCAIView (knowledge discovery framework). Retrieved biomedical texts are ranked based on the frequency of co-occurrences of terms associations included within them.
- ***AEGDA [36]***: Bhasuran and Natarajan [36] proposed a gene-disease association method based on supervised machine learning. For easy reference, we are going to name the method AEGDA "**A**utomatic **E**xtraction of **G**ene-**D**isease **A**ssociations”. The method adopts local and global semantics and syntax techniques for extracting gene-disease associations from the literature. It employs SVM classifier and ensemble learning.
- ***BeFree [37]:*** Bravo et al. [37] proposed a supervised learning-based method called BeFree for identifying gene-disease associations extracted from biological texts. The method employs machine learning techniques coupled with dependency kernel and text’s morpho-syntactic features.
- ***PKDE4J [38]:*** Song et al. [38] proposed the text-mining system PKDE4J, which is an extension of the Stanford CoreNLP [40]. It employs rule-based relation and dictionary-based entity extraction methodology.

Below are brief descriptions of the four gold standard corpora used in the evaluations of AEGDA, BeFree, and PKDE4J:

- ***EU-ADR [41]:*** It is a multi-relation annotated corpus. The corpus is annotated with multiple concepts (e.g., diseases, genes, drugs) and their interrelationships.
- ***GAD [42]:*** The corpus was released as a part of the BeFree system [37]. The corpus focuses solely on the extraction of gene-disease associations including a large number of false, positive, and positive negative associations.
- ***CoMAGC [43]:*** It is a multi-faceted relation annotation corpus. The corpus focuses on gene-cancer associations as well as the frequency of their co-occurrences. Specifically, the corpus focuses on breast, prostate, and ovarian cancers.
- ***PolySearch [44]:*** The corpus was released as a part of the PolySearch system [45] for extracting the associations between over ten biological concepts.

Since AEGDA and BeFree have reported results, we only ran SDDP and SCAIView against the EU-ADR, GAD, CoMAGC, and PolySearch gold standard corpora. Table 4 presents description of the corpora.

**Table 4:** Description of the corpora used in the evaluation.

	EU-ADR	GAD	CoMAGC	PolySearch
Number of abstracts	100	5330	408	374
Number of occurred diseases	964	5330	821	522
Number of unique diseases	126	923	3	10

**Fig 8:**
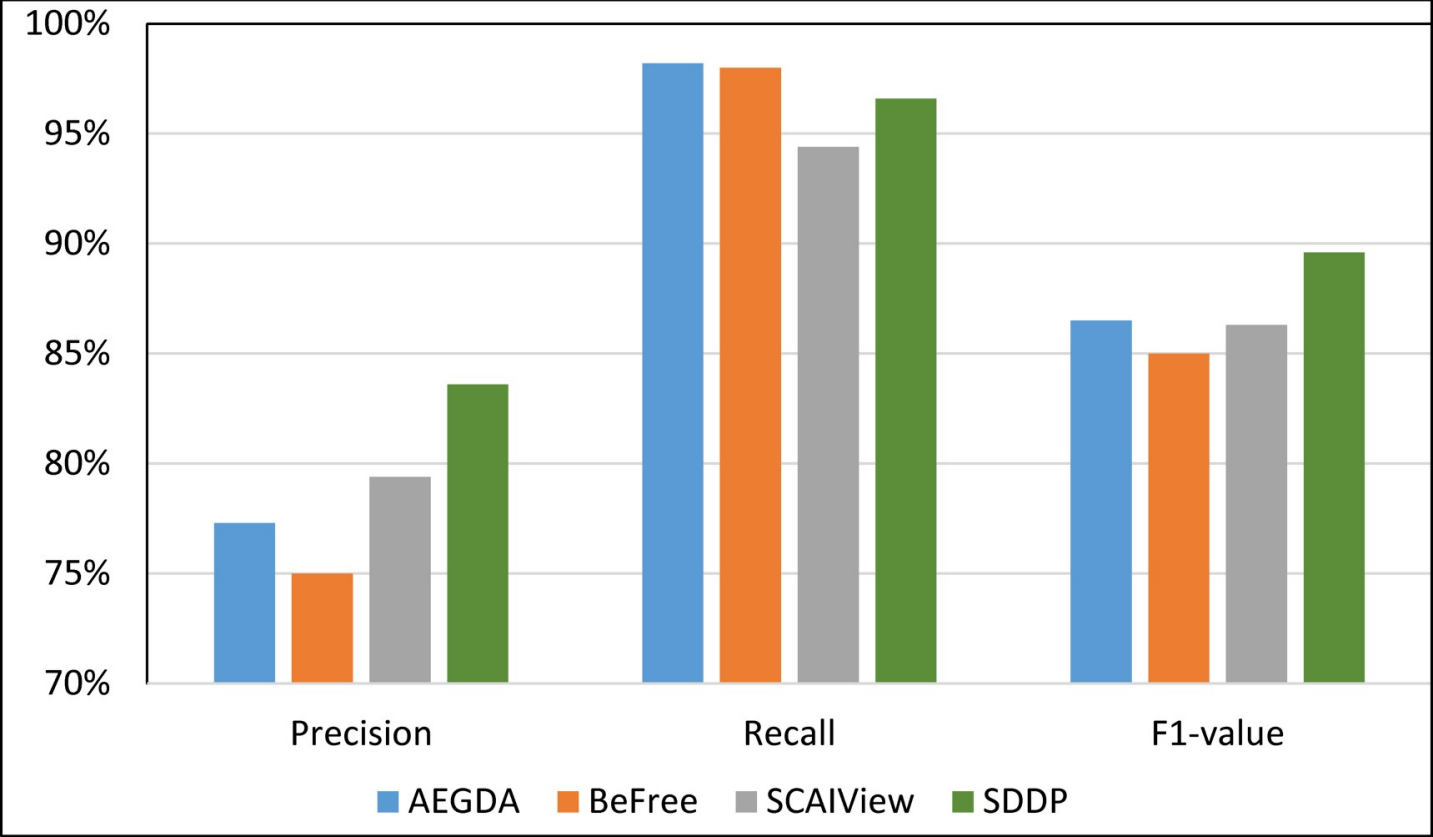
Comparing the performance of the four methods for extracting information pertaining gene-disease associations from the EU-ADR corpus.

**Fig 9:**
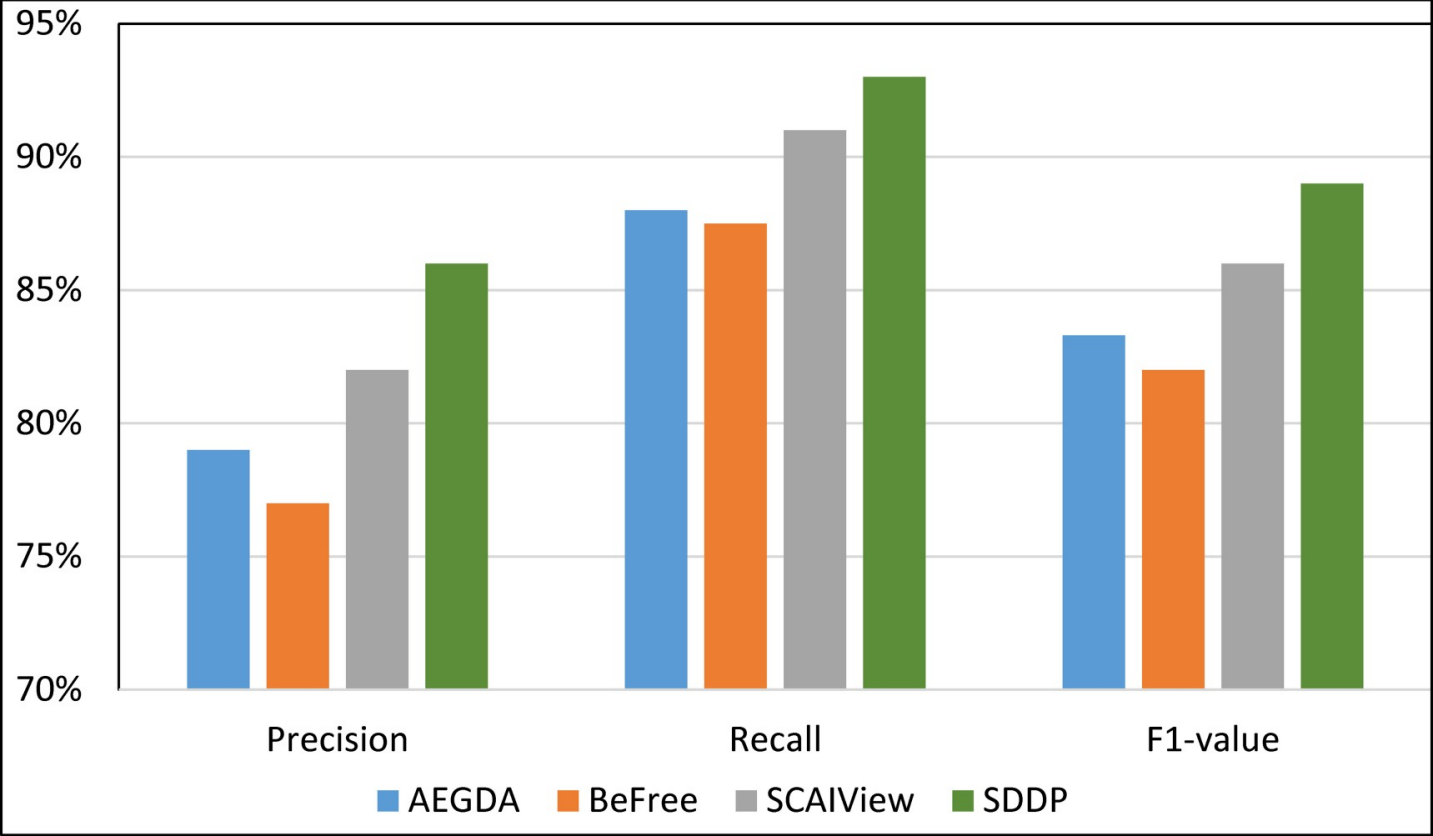
Comparing the performance of the four methods for extracting information pertaining gene-disease associations from the GAD corpus.

**Fig 10:**
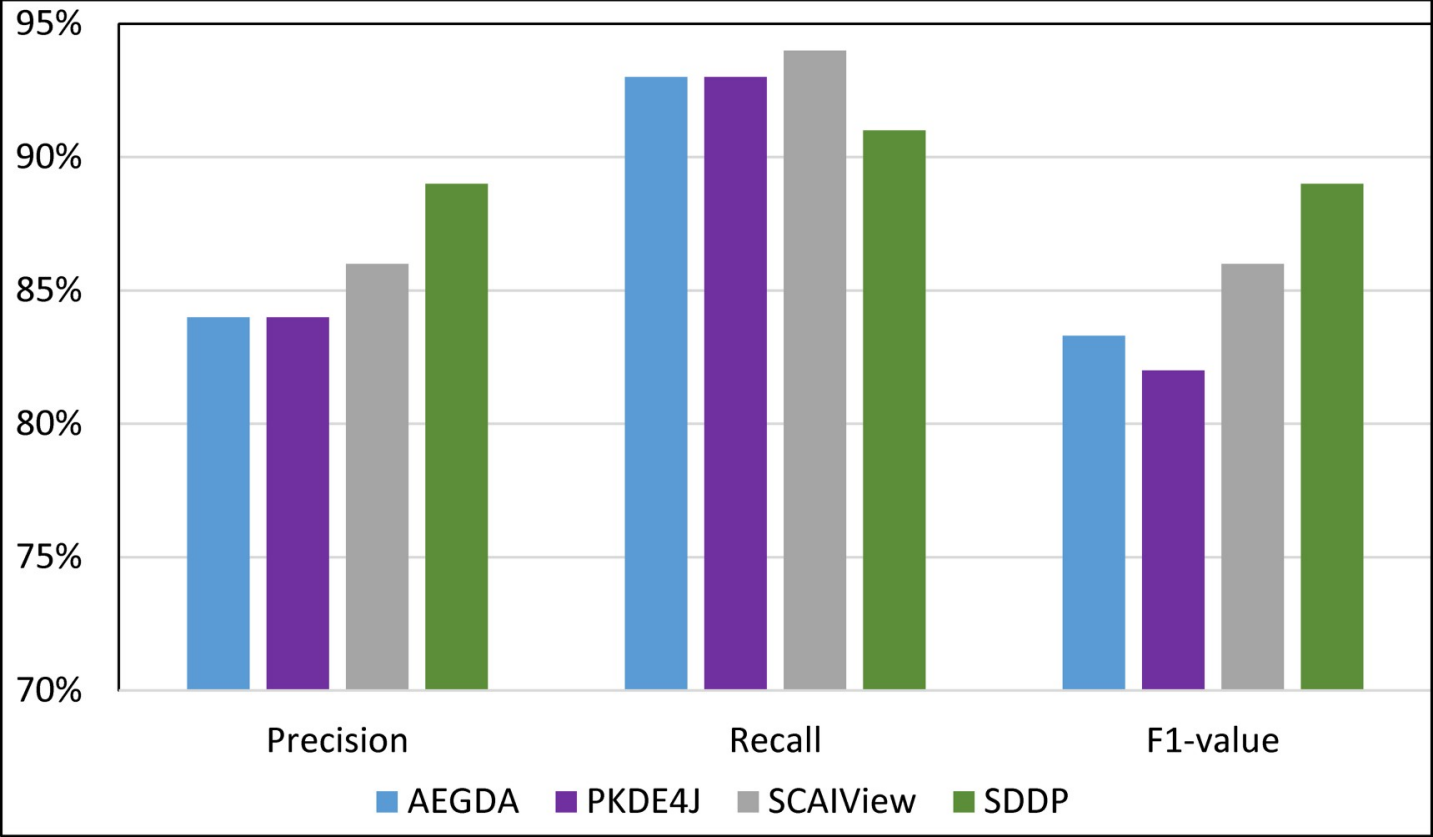
Comparing the performance of the four methods for extracting information pertaining gene-disease associations from the CoMAGC corpus.

**Fig 11:**
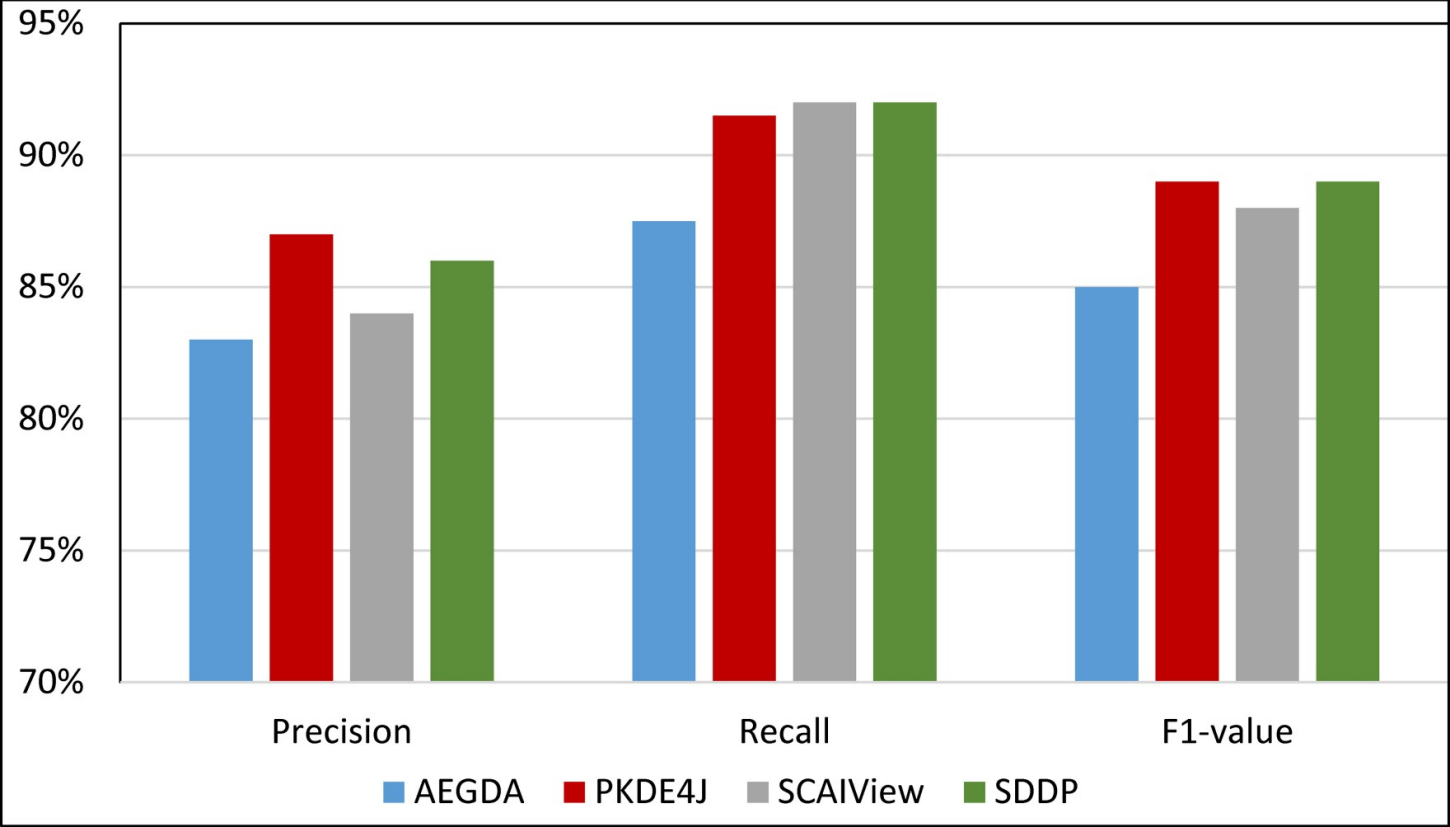
Comparing the performance of the four methods for extracting information pertaining gene-disease associations from the PolySearch corpus.

We computed the Recall, Precision, and F-value of SDDP and SCAIView using the following standard formulas: Recall = TP/(TP+FN); Precision = TP/(TP+FP); F-value = (2 Precision * Recall)/(Precision + Recall), where: FP = False positive, TP = True Positive, and FN = False Negative. Figs 8–11 plots the Precision, Recall, and F1-value using the EU-ADR, GAD, CoMAGC, and PolySearch, respectively.

**Fig 12:**
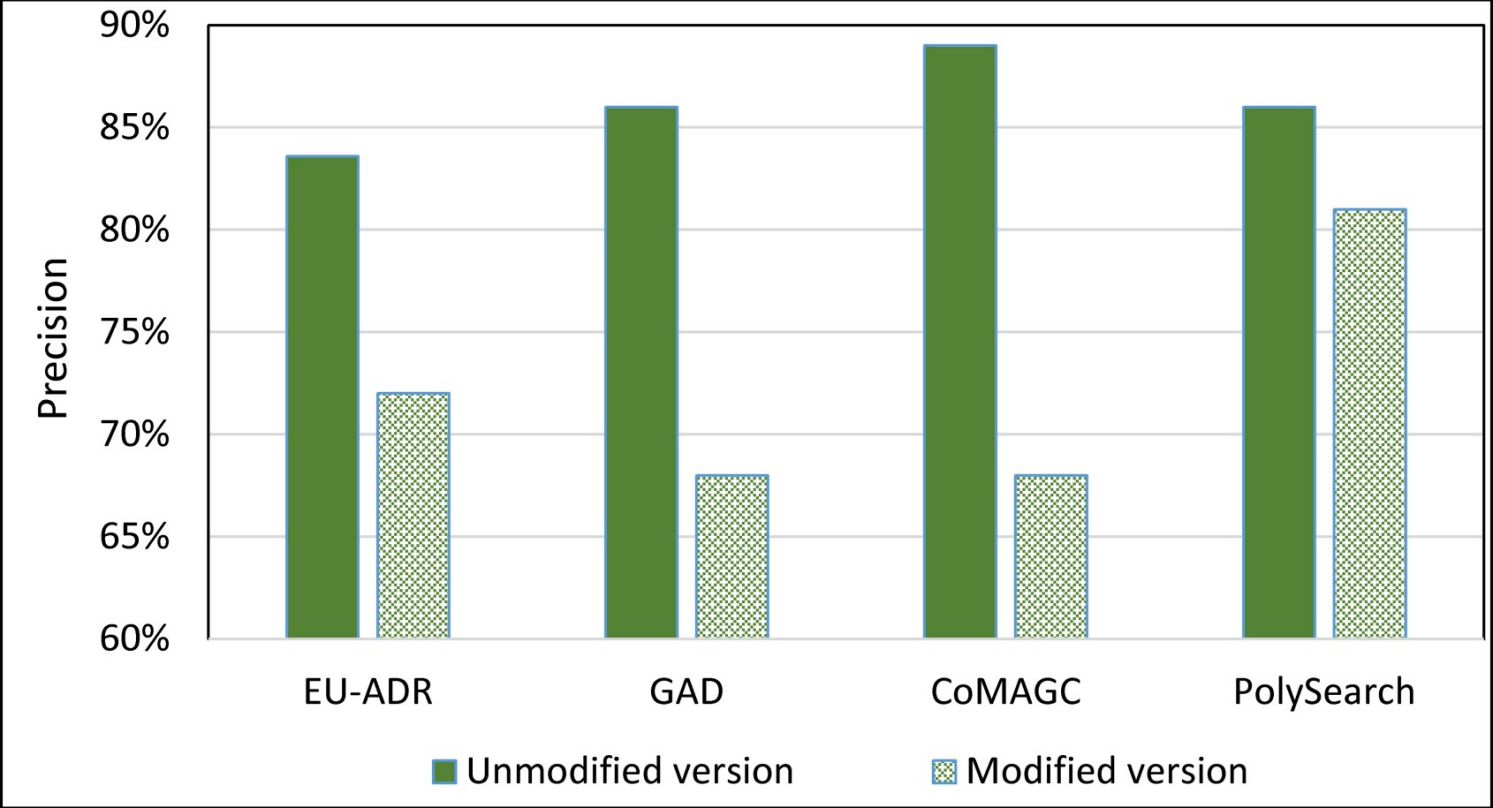
The *overall average* Precision of the unmodified and modified versions of SDDP.

**Fig 13:**
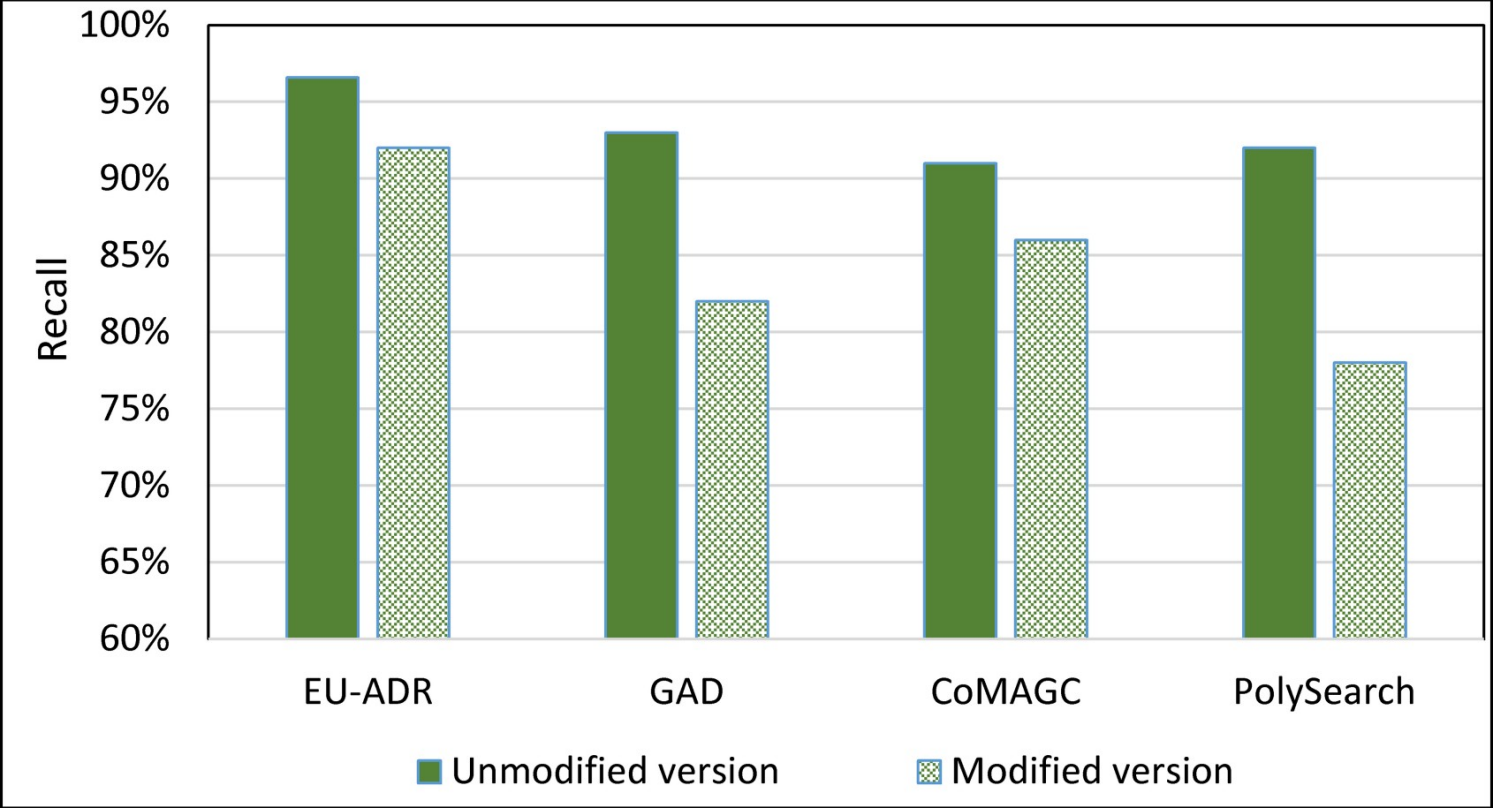
The *overall average* Recall of the unmodified and modified versions of SDDP.

To further evaluate the impact of the information extractor component of SDDP on its prediction accuracy, we constructed a version of SDDP, whose information extractor component is replaced by PolySearch2 [45]. That is, the modified version of SDDP employs PolySearch2 rather than SDDP’s information extractor component for extracting genes and diseases terms from the EU-ADR, GAD, CoMAGC, and PolySearch gold standard corpora. PolySearch2 is a text mining tool for extracting relationships between genes, diseases, drugs, mutations, and metabolites found within texts [45]. PolySearch2 supports generalized information extraction queries. For example, a query could be: For a given term *x*, extract each term *y* associated with *x* from biomedical publications, where *x* and *y* have significant co-occurrences in the publications. We ran the modified version against the four corpora described previously. Then, we compared the prediction accuracies of the modified and unmodified versions of SDDP. Figs 12 and 13 show the results.

As Figs 8–11 show, SDDP outperformed SCAIView, AEGDA, BeFree, and PKDE4J. The experimental results revealed also that the unmodified version of SDDP outperformed the modified one, especially in terms of precision (recall Fig 12). This is attributed to the effectiveness of the strict linguistic rules employed by the information extractor component in extracting not only explicitly mentioned molecular terms (e.g., genes) within texts, but also implicitly mentioned ones. Some important terms pertaining biological molecules and diseases may occur implicitly within biomedical texts. For instance, the nouns “p14ARF gene” and “p53 protein” in Example 8 in Appendix 8 of the Supplemental Material in S1 File will be determined by SDDP to be implicitly associated. Thus, information extraction techniques that rely only on explicitly mentioned terms can miss such vital implicitly mentioned terms. So, the employment of SDDP to the concept of *semantic relationship* between molecular terms in sentences contributed to its performance over the other methods. This concept ensures each co-occurrence of a pair of molecule terms in a sentence is disregarded, if the pair is unrelated grammatically (recall Section “Information Extractor” and the Supplemental material). That is, SDDP considers the co-occurrence of a pair molecule terms in a sentence a reflection of their association only if the pair is semantically related in the sentence.

## Evaluating the risk indicator component of SDDP experimentally

In this test, we aim at evaluating the risk indicator component of SDDP experimentally. Unfortunately, we could not find an accessible comparable method that produces risk indicators. Therefore, we decided to evaluate and compare only the ranking feature of the risk indicator component. Since the ranking feature of SDDP’s risk indicator component plays a significant role in the performance of the component, evaluating it sheds a light on the effectiveness of the whole component. Towards this, we evaluate the ranking feature of SDDP by comparing it experimentally with PWK [46]. The code of the PWK is available at [47].

PWK predicts gene-disease associations by computing the cosine similarity between vectors representing genes and vectors representing a disease. The method assigns vectors to gene and disease terms based on their co-occurrences in PubMed database. The gene-disease associations are predicted with reference to MeSH database [39]. Based on the cosine similarities between genes and a disease, genes are ***ranked*** accordingly.

For the evaluations, we used the data of gene-disease associations in OMIM database [27,28,48] as gold standard. Gene-disease associations in OMIM are manually curated. We selected 3318 genes and 447 diseases that are found also in the MeSH database. We constructed the disease and gene dictionary according to MeSH. We retrieved the biomedical literature associated with the selected genes and diseases terms from PubMed [25]. The date of the search was May 17, 2020. This resulted in 714,214 publications. We submitted the following PubMed query:

(“diseases” [MeSH Terms]) AND “genes” [MeSH Terms] AND (has abstract [text]) AND (English [lang]) AND “humans” [MeSH Terms] AND (“0001/01/01” [PDAT]: “2020/05/17” [PDAT]).

### Evaluating the risk indicator of SDDP in terms of its ranking feature

**Fig 14:**
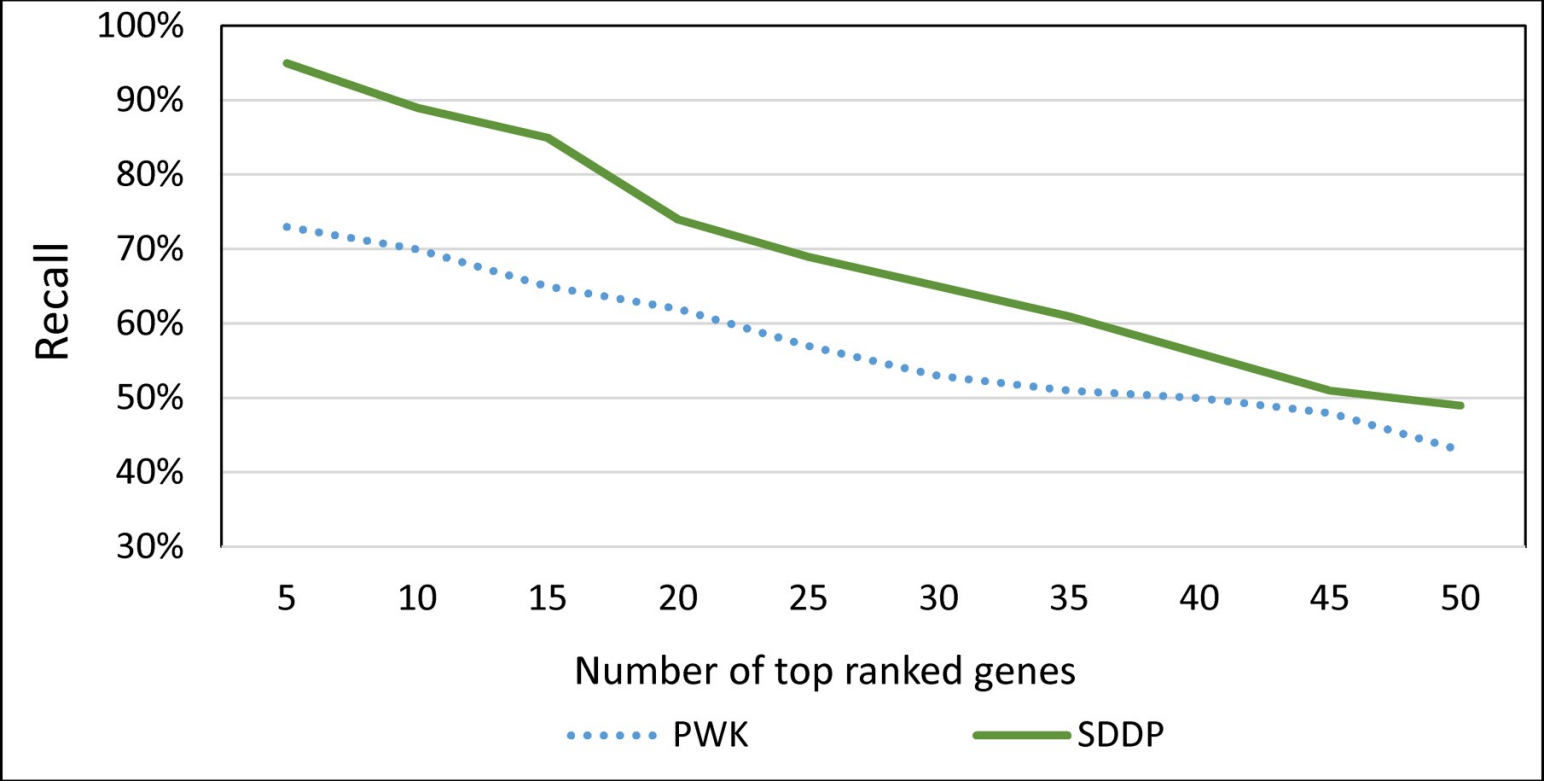
The average Recall of top *x* genes associated with a disease ranked by SDDP and PWK, where *x* ranges from 5–50 in an increment of 5.

For PWK, we ranked genes according to their similarity to a disease by varying the cosine similarity in the range [0–1]. Recall that PWK ranks genes based on their cosine similarities with a disease. For SDDP, we ranked genes as follows. Let *M* be the set of ranked MP combinations associated with a disease output by SDDP’s Risk Indicator component. Let *r* be the dominance rank of combination *c* ∈ *M* (recall Section “Risk Indicator”). Each gene ∈ *c* is assigned the rank of the dominance rank of *c*. We identified the top *x* genes ranked by SDDP and PWK, where *x* ranges from 5–50 in an increment of 5, *with reference to the OMIM gold standard*. Fig 14 shows the result.

As Fig 14 shows, SDDP outperformed PWK. This can reflect the practical viability and effectiveness of SDDP’s combination of implicit and explicit techniques. The implicit technique is SDDP’s feature of inferring molecule terms (e.g., genes) that co-occur *implicitly* with disease terms using the rules of predicate logic. The explicit technique is SDDP’s feature of extracting molecule terms that co-occur *explicitly* with disease terms in texts using the methodology described in Section Information Extractor. That is, the performance of SDDP is attributed, in part, to the effectiveness of the rules of predicate logic in inferring MP terms that co-occur *implicitly* with disease terms in biomedical texts. Moreover, the employment of SDDP to the concept of identifying *dominant* MPs associated with a disease terms (recall Section Risk Indicator) has also contributed significantly to the performance of SDDP. This concept guarantees that uninformative MPs associated with a disease term in texts are filtered and excluded. A MP associated with a disease is considered uninformative, if it has only few occurrences in abstracts or found in abstracts associated with many other diseases.

Over all, we attribute the performance of SDDP over PDW to the fact that SDDP employs a combination of statistical and logic-based techniques while PWK employs only a statistical-based technique. That is, SDDP includes a combination of statistical-based *explicit* term extraction as well as and logic-based *implicit* term extraction techniques. Our hypothesis is that crucial molecule terms and disease terms are likely to have *implicit* co-occurrences in biomedical texts. Thus, systems that employ only statistical-based techniques (such as PWK) are likely to miss identifying vital molecule-disease association information.

### Evaluating the impact of the size of retrieved texts on the ranking accuracy of SDDP

In real-world setting, the size of biomedical literature increases constantly over time. Therefore, it is important for evaluating the impact of accumulating size of biomedical literature dataset on the prediction accuracy of SDDP. Towards this, we evaluated the prediction accuracies of SDDP and PWK using different sizes of the dataset. We partitioned the dataset into four disjoint testing subsets at random. We then performed four evaluation runs over the set of testing dataset, which accumulates in each run successively. Initially, the two systems were run against one of the four subsets. At each run, thereafter, an unused subset is added to the current testing subset, and the systems are run against the accumulating set. Thus, the set of dataset accumulates successively.

**Fig 15:**
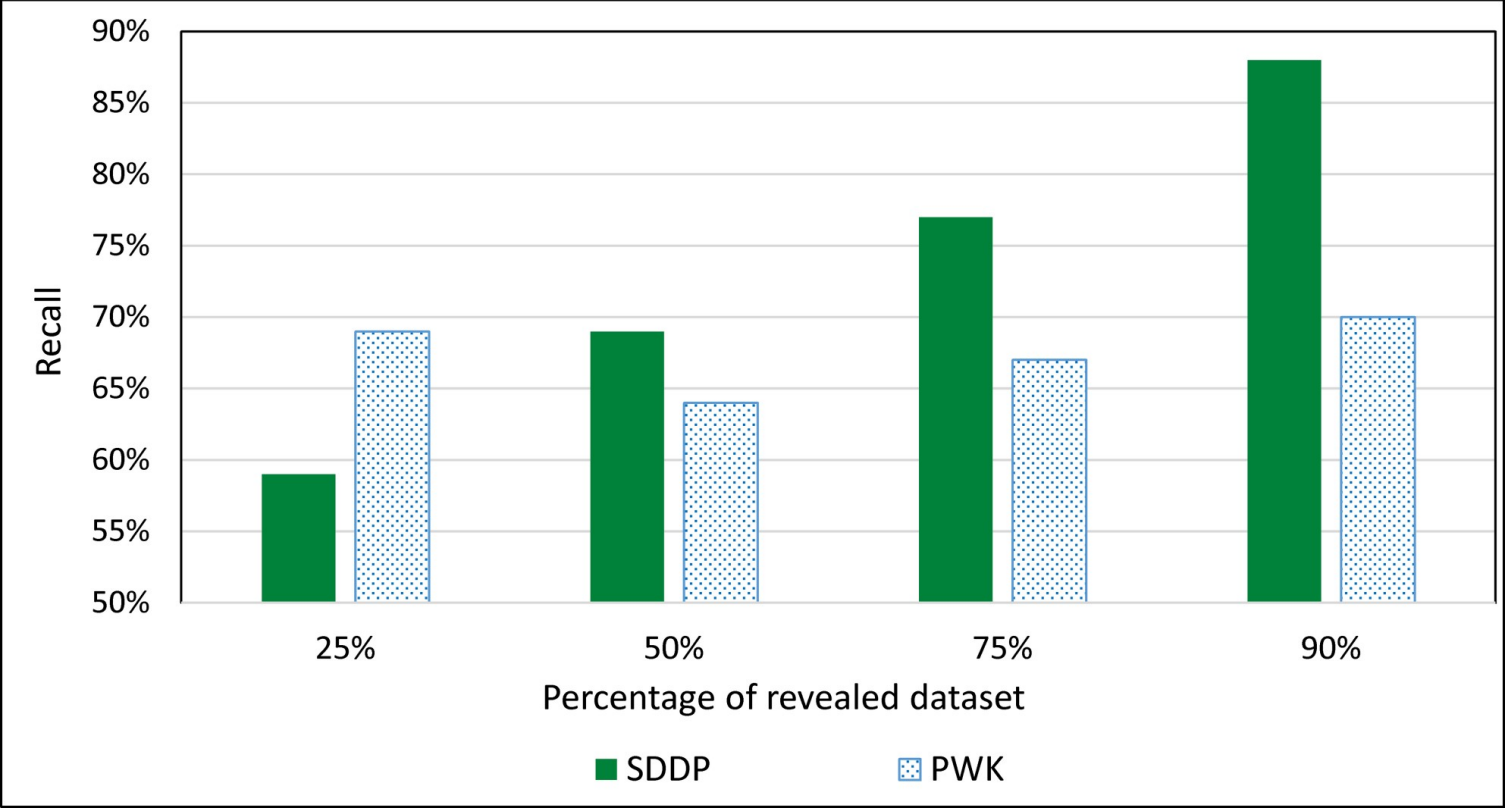
Average Recall of ranking the top 5 genes associated with a disease based on accumulating size of dataset.

For PWK, we considered 0.6 as the cosine similarity for ranking genes according to their similarity to a disease. This is because: (1) the recall rate of PWK decreases as cosine similarity increases and its precision rate increases as cosine similarity increases, and (2) the recall and precision tend to remain stable around 0.6. We evaluated the prediction accuracies of the two systems using different sizes of the dataset. Fig 15 shows the average Recall of ranking the top 5 genes associated with a disease based on accumulating size of revealed PubMed texts.

The experimental results showed that the prediction accuracy of the SDDP improved constantly as the size of accumulating revealed dataset increased (see Fig 15). After the size of accumulating revealed dataset reached 33%, the prediction accuracy of PWK improved constantly as the size of accumulating dataset increased. However, the rate at which the prediction accuracy of SDDP increased was higher than that of PWK. This is advantageous to SDDP, since the size of biomedical literature associated with biomarker molecules increases constantly over time in real-world setting. This was confirmed by the experimental results, where the set of mutated genes/MMs associated with a specific disease that was inferred by SDDP increased as the fraction of revealed literature dataset increased. This in turn, led to a continuous enhancement of the MPIN, which represents the hierarchical interrelationships between the MPs of the disease. The reason for the constant improvement of the prediction accuracy of SDDP as the size of dataset increases is that every time a new set of texts is revealed, new MMs are extracted by the Information Extractor component. This leads to optimizing and enhancing current MCTs as well as the construction of new MCTs rooted at the newly extracted MMs. The enhancement of MCTs, in turn, leads to the enhancement of MPINs. The enhancement of MPINs, in turn, leads to inferring new MPs. Inferring New MPs, in turn, may lead to updating and optimizing the set of dominant MPs associated with a disease.

## Conclusion

We proposed in this paper a novel methodology for personalizing an individual’s degree of future susceptibility to a specific disease. We implemented the methodology in a working system called SDDP. To the best of our knowledge, this is the first research work that combines the following three techniques for predicting an individual’s degree of future susceptibility to a specific disease: information extraction, inference rules of predict logic, and modeling the interrelationships among the molecular pathways of a specific disease. Moreover, this is the first research work, to the best of our knowledge, that employs the inference rules of predict logic to infer as many as possible undetected molecular pathways of a disease for an individual based on a few molecular pathways, to which traceable biomarkers detected from the individual belong.

The logic-based inference component of SDDP ensures that the *collective combination* of inferred molecular pathways of a disease for an individual, whose traceable biomarkers were detected from the individual, is likely be an indicative of the disease. We evaluated the information extraction feature of SDDP by comparing it with the following four text mining methodologies: SCAIView [24], AEGDA [36], BeFree [37], and PKDE4J [38]. The experimental results showed that SDDP outperformed the other methods. This is attributed to the effectiveness of the strict linguistic rules employed by the information extractor component of SDDP. That is, the strict linguistic rules employed by SDDP contributed to its performance.

We also evaluated the ranking feature of SDDP by comparing it experimentally with PWK [46]. The results showed that SDDP outperformed PWK, which is attributed, mainly, to its ability to infer molecular pathways that co-occur *implicitly* with disease terms using the rules of predicate logic. The experimental results revealed that the performance of SDDP over PWK kept increasing at a higher rate as the size of dataset kept being increased. This is advantageous to SDDP, since the size of biomedical literature associated with MMs increases constantly over time in real-world setting.

## Supporting information

S1 File (PDF)
