# Supplementary material for: Personizing the prediction of future susceptibility to a specific disease
Source: PLoS One. 2021 Jan 6;16(1):e0243127. doi: 10.1371/journal.pone.0243127 (PMC7787538; doi:10.1371/journal.pone.0243127)
Supplement: S1 File — (PDF) [file pone.0243127.s001.pdf]

<u>Supplemental Material</u>

Appendix A

<u>Illustration of our proposed linguistic rules</u>

In each of the next two subsections, we illustrate our proposed rules using sentences extracted from biomedical literature. In these examples, we show how the semantic relationships between biomedical nouns can be determined using our proposed rules. We divide each sentence into simple sentences using dependency grammar. Each simple sentence is an independent clause, which contains a subject and a predicate. We place each independent clause inside a rectangle for easy reference. In each example, the words that comprise a sentence are tagged as follows: (N) for noun, (V) for verb, (PREP) for preposition, and (PRON) for pronoun.

Sentences Containing Preposition Modifiers

Our first proposed semantic rules are based on the following linguistics observations [1, 2]: (1) two independent clauses connected by a preposition modifier (such as "but", "while", and "whereas") are usually unrelated, and (2) all nouns within an independent clause are usually related. The following are our proposed rules, which are based on the above observations:

1. The co-occurrence of a pair of molecule terms in a sentence is considered semantically unrelated, if the two terms occur in two different independent clauses connected by a preposition modifier. This is because the two terms do not have dependency relationship in this case.
2. The co-occurrence of a pair of molecule terms within an independent clause (i.e., inside a rectangle in our examples) is considered semantically related.

In Examples 1-4, we demonstrate how these semantic rules conform to the linguistics theory stated previously. In these sentences, we determine the semantic relationship between each pair of generic biomedical nouns.

Example 1: Consider the following sentence: *"YIL169C is conserved with Chemotaxis while Truncated_TBY is conserved with INTEGRASE"*. The following is the syntactic structure of the sentence in terms of its constituents of independent clauses.

YIL169C (N) **is** (V) **conserved with Chemotaxis** (N),

while (PREP)

Truncated_TBY (N) **is** (V) **conserved with INTEGRASE** (N)

The subject noun of the first independent clause "YIL169C" is semantically related to the noun "Chemotaxis". The subject noun of the second independent clause "Truncated_TBY" is semantically related to the noun "INTEGRASE". However, each of the nouns " YIL169C" and "Chemotaxis" is unrelated to each of the nouns "Truncated_TBY" and "INTEGRASE", because they belong to two different independent clauses connected by the preposition modifier "while".

Example 2: Consider the following sentence: *"SLC6A2 and SLC6A4 are linked in some studies, whereas OCD was linked to G1287A and 5-HIT"*. The following is the syntactic structure of the sentence in terms of its constituents of independent clauses.

SLC6A2 (N) **and SLC6A4** (N) **are** (V) **linked in some studies** (N),

whereas (PREP)

OCD (N) **was** (V) **linked to G1287A** (N) **and 5-HIT** (N)

The nouns "SLC6A2" and "SLC6A4" are semantically related. The noun "OCD" is semantically related to the nouns "G1287A" and "5-HIT". However, each of the nouns "SLC6A2" and "SLC6A4" is unrelated to each of the nouns "OCD", "G1287A", and "5-HIT", because the first and second sets of nouns belong to two different independent clauses connected by the preposition modifier " whereas".

Example 3: Consider the following sentence: *"Alport syndrome is associated with COL4A5, while COLA3 and COLA4 are associated with autosom"*. Below is the syntactic structure of the sentence in terms of its constituents of independent clauses:

Alport syndrome (N) **is** (V) **associated with COL4A5** (N),

while (PREP)

COLA3 (N) **and COLA4** (N) **are** (V) **associated with autosom** (N)

The nouns "Alport syndrome" and "COL4A5" are semantically related. The nouns "COL4A3" and "COL4A4" are semantically related to the noun "autosom". However, each of the nouns "Alport syndrome" and "COL4A5" is unrelated to each of the nouns "COL4A3", "COL4A4", and "autosom", because the two sets of nouns belong to two different independent clauses connected by the preposition modifier "while".

Example 4: Consider the sentence: *"In response to stimulating, Nck1 exhibited decreased CD69 expression but Nck2 exhibited increased CD69 expression"*. Below is the syntactic structure of the sentence in terms of its constituents of independent clauses:

In response to stimulating,

Nck1 (N) **exhibited** (V) **decreased CD69_expression** (N),

but (PREP)

Nck2 (N) **exhibited** (V) **increased CD69_expression** (N)

The nouns "Nck1" and ""CD69 expression" are semantically related. The nouns "Nck2" and ""CD69 expression" are semantically related. However, the nouns "Nck1" and "Nck2" are semantically unrelated, because they belong to two different independent clauses connected by the preposition modifier "but".

Sentences Containing Pronouns Defining Antecedents

According to linguistics, an antecedent noun is usually related to the subsequent noun(s), if the subsequent noun(s) is connected to the antecedent by a pronoun (such as "which", "who", "it", "whom", and "that") [3]. We propose our second semantic rules based on this linguistic observation, as follows:

1. An antecedent noun is semantically related to a subsequent noun(s), if the two nouns are connected by a pronoun. Towards this, we replace each pronoun with the *closest* noun found under the *predecessor independent clause*. This conforms to grammar and linguistics, which treat a pronoun as a word that can be substituted by a noun or noun phrase. In Examples 5-9, we strikethrough each pronoun and replace it with the *closest* noun found under the predecessor independent clause.
2. An explicit or implicit pronoun preceded by a conjunction (i.e., "and" and "or") refers to the *subject* of closest predecessor independent clause. In Examples 5-9, we strikethrough each pronoun preceded by a conjunction and replace it with the *subject* of closest predecessor independent clause. In the case of an *implicit* pronoun preceded by a conjunction, we also replace it with the *subject* of closest predecessor independent clause.

For the sake of clarification, we perform the following in Examples 5-9:

1. We type the subject of the first independent clause using a different font.
2. We type each noun that replaces a pronoun: (1) in italics, (2) in a different font, and (3) place quotation marks around it. The replacement noun plays the role of the subject of the independent clause that comes immediately after the pronoun.

In Examples 5-9, we demonstrate how these semantic rules conform to the linguistics theory stated above. We determine the semantic relationship between each pair of generic biomedical nouns. Recall that all nouns (including the replacement nouns) within an independent clause are related.

Example 5: Consider the sentence: *"The two variants of Hemoglobin are HbSS which causes death, and HbAS which protects against malaria"*. The following is the syntactic structure of the sentence in terms of its constituents of independent clauses.

The two variants of Hemoglobin (N) **are** (V) **HbSS** (N)

~~**which**~~ (PRON) "*HbSS*" **causes** (V) **death** (N) **and HbAS** (N),

~~**which**~~ (PRON) "*HbAS*" **protects** (V) **against malaria** (N)

The subject noun "Hemoglobin" is semantically related to the noun "HbSS". In the second independent clause, the pronoun "which" is replaced by the *closest* noun found under the predecessor independent clause (i.e., "HbSS"), which becomes the subject of the second independent clause. Therefore, the nouns "HbSS" is semantically related to the nouns "death" and "HbAS". In the third independent clause, the pronoun "which" is replaced by the *closest* noun found under the predecessor independent clause (i.e., "HbAS") Therefore, the nouns "HbAS" and "malaria" are semantically related.

Example 6: Consider the following sentence: *"Zbtb7A is a repressor of the tumor suppressor p14ARF that in turn lowers the expression of p53 gene and it is a central regulator in oncogenesis"*. The following is the syntactic structure of the sentence in terms of its constituents of independent clauses.

Zbtb7A (N) is (V) a repressor of the tumor suppressor p14ARF (N)

~~that~~ "*p14ARF*" in turn lowers (V) the expression of p53 gene (N)

and ~~it~~ *p14ARF*" is (V) a central regulator in oncogenesis (N).

The subject noun "Zbtb7A" is semantically related to the noun "p14ARF". The pronoun "that" is replaced by the *closest* noun found under the predecessor independent clause (i.e., the noun "p14ARF"), which becomes the subject noun of the second independent clause. Therefore, the nouns "p14ARF" and "p53 gene" are semantically related. Since the pronoun "it" follows the conjunction "and", it is replaced by the *subject* noun of the closest predecessor independent clause (i.e., the noun "p14ARF"), which becomes the subject of the third independent clause. Therefore, the nouns "p14ARF" and "oncogenesis" are semantically related.

Example 7: Consider the following sentence: *"p16 is a cell-cycle protein, which interacts with the sequester MDM2, and it inhibits the ability of CDK4 to interact with cyclins D"*. The following is the syntactic structure of the sentence in terms of its constituents of independent clauses.

p16 (N) is (V) a cell-cycle protein (N),

~~which~~ (PRON)"*cell-cycle protein*" interacts(V)with the sequester MDM2 (N),

and ~~it~~ "*cell-cycle protein*" inhibits (V) the ability of CDK4 (N)

to interact (V) with cyclins D (N).

The subject "p16" is semantically related to the noun "cell-cycle protein". The pronoun "which" is replaced by the *closest* noun under the predecessor independent clause (i.e., the noun "cell-cycle protein"), which becomes the subject of the second independent clause. Therefore, the nouns "cell-cycle protein" and "MDM2" are semantically related. Since the pronoun "it" follows the conjunction "and", it is replaced by the *subject* noun of the closest predecessor independent clause (i.e., the noun "cell-cycle protein"), which becomes the subject of the third independent clause. Therefore, the nouns "cell-cycle protein" and "CDK4" are semantically related.

Example 8: Consider the following sentence: *"p14ARF gene inhibits mdm2 protein and promotes p53 protein which promotes p21 protein which binds and inactivates cyclin-CDK".*

p14ARF gene (N) inhibits (V) mdm2 protein (N)

and "*p14ARF gene*" promotes (V) p53 protein (N)

~~which~~ (PRON) "*p53 protein*" promotes (V) p21 protein (N)

~~which~~ (PRON) "p21 protein" binds (V)

and "*p21 protein*" inactivates (V) cyclin-CDK (N).

The subject noun "p14ARF gene" is semantically related to the noun "mdm2 protein". In the second independent clause, the *implicit pronoun* that follows the conjunction "and" is replaced by the *subject* noun of the closest predecessor independent clause (i.e., the noun "p14ARF gene"), which becomes the subject of the second independent clause. Therefore, the nouns "p14ARF gene" and "p53 protein" are semantically related. In the third independent clause, the pronoun "which" is replaced by the *closest* noun found under the predecessor independent clause (i.e., "p53 protein"), which becomes the subject of the third independent clause. Therefore, the nouns "p53 protein" and "p21 protein" are semantically related. In the fourth independent clause, the pronoun "which" is replaced by the *closest* noun found under the predecessor independent clause (i.e., "p21 protein"), which becomes the subject of the fourth independent clause. In the fifth independent clause, the *implicit pronoun* that follows the conjunction "and" is replaced by the *subject* noun of the closest predecessor independent clause (i.e., "p21 protein"), which becomes the subject of the fifth independent clause. Therefore, the nouns "p21 protein" and "cyclin-CDK" are semantically related.

Example 9: Consider the following sentence: *"Zbtb7A protein binds to HIV type I and interacts with BCL-6".*

Zbtb7A protein (N) binds (V) to HIV type I (N)

and "*Zbtb7A protein*" interacts (V) with BCL-6 (N)

The subject noun "Zbtb7A protein" is semantically related to the noun "HIV type I". The *implicit pronoun* that follows the conjunction "and" is replaced by the *subject* noun of the closest predecessor independent clause (i.e., the noun "Zbtb7A protein"), which becomes the subject of the second independent clause. Therefore, the nouns "Zbtb7A protein" and "BCL-6" are semantically related.

Appendix B

A complete set of inference rules for T2D.

Table 1 shows the complete set of inference rules for T2D. Table 2 shows abbreviation of the terms used in Table 1.

Table 1: A complete set of inference rules for T2D. MP_x denotes Diabetes Molecular Pathway *x*

R_1: $(MP_{SMB} \wedge MP_{CTM}) \rightarrow (MP_{TRXN} \rightarrow MP_{ATM})$
R_2: $(MP_{CTM} \wedge MP_{ATM}) \rightarrow (MP_{RRR} \rightarrow MP_{TRAN})$
R_3: $MP_{RRR} \rightarrow (MP_{TRAN} \rightarrow (MP_{PM} \vee MP_{ST}))$
R_4: $(MP_{IIT} \vee MP_{MB}) \rightarrow MP_{CTM}$
R_5: $MP_{SMB} \rightarrow MP_{TRXN}$
R_6: $((MP_{IIT} \wedge MP_{MB}) \vee MP_{TRAN}) \rightarrow MP_{ST}$
R_7: $MP_{CTM} \rightarrow MP_{RRR}$
R_8: $MP_{TRAN} \rightarrow (MP_{PM} \vee MP_{ST})$
R_9: $MP_{MIT} \rightarrow (MP_{GPLD1} \rightarrow MP_{TRAN})$
R_{10}: $(MP_{MIT} \wedge MP_{TRXN}) \rightarrow (MP_{LTP} \rightarrow MP_{ATCC})$
R_{11}: $MP_{THBS1} \rightarrow (MP_{ST} \rightarrow MP_{ATCC})$
R_{12}: $MP_{CCR} \rightarrow MP_{IMM}$
R_{13}: $(MP_{LDLR} \wedge MP_{CEM}) \vee (MP_{ATCC} \wedge MP_{BCDP}) \rightarrow MP_{APO}$
R_{14}: $(MP_{LF} \wedge MP_{ATCC}) \rightarrow (MP_{CCR} \rightarrow MP_{THBS1})$
R_{15}: $(MP_{LF} \wedge MP_{MIT}) \rightarrow (MP_{ST} \rightarrow MP_{ATCC})$
R_{16}: $MP_{CCR} \rightarrow MP_{FABP}$
R_{17}: $MP_{CCR} \vee MP_{LDR} \rightarrow MP_{GPLD1}$
R_{18}: $(MP_{MIT} \vee MP_{LDLR}) \rightarrow MP_{APO}$
R_{19}: $(MP_{ATCC} \wedge MP_{BCDP}) \rightarrow MP_{CD28}$
R_{20}: $(MP_{TAA} \wedge MP_{TRXN}) \rightarrow MP_{IR}$
R_{21}: $MP_{ATCC} \rightarrow (MP_{SMP} \rightarrow MP_{ST})$
R_{22}: $MP_{CTLA\text{-}4} \rightarrow MP_{ICOS}$
R_{23}: $MP_{TAA} \rightarrow (MP_{IR} \vee MP_{PM})$
R_{24}: $MP_{VHD} \rightarrow MP_{LGF}$
R_{25}: $MP_{APO} \rightarrow (MP_{LDLR} \vee MP_{CEM})$
R_{26}: $MP_{TGL} \rightarrow (MP_{LF} \vee MP_{IMM})$
R_{27}: $MP_{GPLD1} \rightarrow MP_{ATCC}$
R_{28}: $MP_{SMP} \rightarrow MP_{ST}$
R_{29}: $MP_{THBS1} \rightarrow MP_{GPLD1}$
R_{30}: $MP_{CTLA\text{-}4} \rightarrow MP_{CD28}$
R_{31}: $MP_{ICOS} \rightarrow (MP_{CD28} \vee MP_{CTLA\text{-}4})$

Table 2: Abbreviation of the terms used in Table 1

MP Term	Abbreviation
Membrane Biogenesis	MB
Inorganic Ion Trans.	IIT
Carbohydrate Transp. & Metabolites	CTM
Replication, Recombination, Repair	RRR
Aminoacid Trans. & Metabolites	ATM
Signal Transduction	ST
Posttranslational Modification	PM
Transcription	TRXN
Translation	TRAN
Secondary Metabolites Biosynthesis	SMB
Thrombospondin 1	THBS1
Glycosylphosphatidylinositol Specific Phospholipase D1	GPLD1
cytotoxic T-lymphocyte-associated protein 4	CTLA-4
Cluster of Differentiation 28	CD28
Inducible T Cell Costimulatory	ICOS
Adipocyte function and lipid metabolism	ATCC
Cytoesqueleton and extracellular matrix	CEM
Mechanism of insulin in tissue	MIT
Beta cell dysfunction of the pancreas	BCDP
Transporters to aromatic aminoacids	TAA
Cell cycle regulators	CCR
inflammatory response	IR
Liver function	LF
Vascular and hypothalamic dysfunction	VHD
immunoglobulin	IMM
Fatty-acid binding protein	FABP
**triglyceride lipase**	TGL
lipase gene family	LGF
low-density lipoprotein receptor	LDLR
Apolipoproteins	APO
lipid transfer protein	LTP
transmembrane receptors	TRAN

Appendix C

Description of how risk indicators are computed

An indicator value is assigned to a combination of MPs of a disease for an individual as follows. Let S be the set of MPs of a specific disease. We assign a score to each combination $c \subseteq S$. The score reflects the degree of association between the combination c and the disease. Specifically, it reflects the ***dominance*** status of c relative to each other combination $c' \subseteq S$. First, we compute the pairwise *beats* and *looses* for each combination. This is performed based on the co-occurrences of the MPs in the combination in the abstracts of biomedical publications associated with a disease d under consideration. Combination c_i beats combination c_j, if the number of times that the co-occurrence weight of c_i is greater than that of c_j in abstracts. Eventually, each combination c is assigned a score, which is the difference between the number of times that c beats the other combinations and the number of times it loses. This concept is formalized in Definition 1.

Definition 1– A keyword's pairwise score: Let $c_i > c_j$ denote that the number of incidents where the number of occurrences of MP combination k_j is greater than that of combination k_i in the publications associated with a disease under consideration. The pairwise score of combination c_i equals the following:

$$\left|\{c_j \in C : c_i > c_j\}\right| - \left|\{c_j \in C : c_j > c_i\}\right|$$

where C is the set of combinations.

To this end, the combination c_i will be assigned a dominance score S_{c_i}, which is determined as follows.

$$S_{c_i} = N_{beat} - N_{loose}$$

where N_{beat} is the number of times that c_i beat the other combinations (i.e., has the highest occurrence among all other combinations) and N_{loose} is the number of times that c_i lost (i.e., does not have the highest occurrence).

If we sum the dominance scores of all combinations, we find that the result is zero. The highest possible score is (t - 1) and the lowest possible score is -(t - 1), where t is the number of combinations. Finally, the combinations are ranked based on their dominance scores.

Example 1: Consider that there are 10 combinations of MPs: c_1-c_{10}. Consider that the number of co-occurrences of each of the 10 combinations in 3 biomedical publications (p_1-p_3) associated with the disease under consideration is as shown in Table 1. Table 2 shows how the score S_t of each of the 10 combinations is computed based on its number of occurrences in the 3 publications presented in Table 1. For example, let c_9 be the combination of MPs, to which detected **traceable biomarker**s from an individual belong. The individual will be given the risk indicator 3.

Table 1: The number of co-occurrences of each of the 10 MP combinations in 3 publications associated with a disease as described in Example 1

	c_1	c_2	c_3	c_4	c_5	c_6	c_7	c_8	c_9	c_{10}
p_1	3	0	0	0	7	0	3	6	3	0
p_2	0	3	7	3	0	0	0	3	0	4
p_3	3	3	5	0	0	6	0	0	4	0

Table 2: Beats/looses scores of the combinations of the MPs described in Example 1 based on their number of co-occurrences on the 3 publications as shown in Table 1

	c_1	c_2	c_3	c_4	c_5	c_6	c_7	c_8	c_9	c_{10}
c_1	0	0	+	-	0	0	-	+	+	-
c_2	0	0	+	-	-	0	-	0	+	0
c_3	-	-	0	-	-	0	-	-	-	-
c_4	+	+	+	0	0	0	0	+	+	+
c_5	0	+	+	0	0	0	-	0	0	0
c_6	0	0	0	0	0	0	0	+	0	0
c_7	+	+	+	0	+	0	0	+	+	0
c_8	-	0	+	-	0	-	-	0	-	0
c_9	-	-	+	-	0	0	-	+	0	-
c_{10}	+	0	+	-	0	0	0	0	+	0
S_{c_i}	0	+1	+8	-6	-1	-1	-6	+4	+3	-2
**Risk Indicator**	**5**	**4**	**1**	**9**	**6**	**6**	**9**	**2**	**3**	**8**

"+" denotes: combination c_i beat combination c_j. "-" denotes: combination c_i lost to combination c_j. "0" denotes: c_i and c_j have the same number of beats and looses. S_{ci} is the dominance score of c_i.

Illustration that shows how Table 2 is filled out:

We show below how the fields (g_3, g_1), (g_4, g_1), and (g_1, g_2) are filled out:

- The field (g_3, g_1) is assigned the symbol "**-**" because: g_1 beat g_3 one time and it lost to g_3 two times.
- The field (g_4, g_1) is assigned the symbol "**+**" because: g_4 beat g_1 two times and it lost to g_1 one time.
- The field (g_1, g_2) is assigned the symbol "**0**", because: g_1 beat g_2 one time and it lost to g_2 one time.

Illustration that shows how the dominance scores "S_c" in Table 2 are calculated:

S_{ci} = Number of "+" of c_i **-** Number of "-" of c_i.

We show below how dominance scores of c_2, c_3, c_5, c_8, and c_1.

- $S_{c_2} = 3 - 2 = \mathbf{+1}$
- $S_{c_3} = 8 - 0 = \mathbf{+8}$
- $S_{c_5} = 1 - 2 = \mathbf{-1}$
- $S_{c_8} = 5 - 1 = \mathbf{+4}$
- $S_{c_1} = 3 - 3 = \mathbf{0}$

An individual is given a risk indicator value that reflects his/her future degree of susceptibility to the disease *d* as follows. Let c_x be the combination of MPs output by component Logic Inferencer for the individual (recall Section "Logic Inferencer"). The individual will be assigned a risk indicator value corresponds to the dominance rank of combination c_x. That is, after all MP combinations are ranked based on their dominance scores, the individual will be assigned a risk indicator value corresponds to the dominance rank of c_x.

References

[1]. Karttunen, Lauri. 1976. Discourse Referents. in J. McCawley (ed.) *Syntax and Semantics 7: Notes From the Linguistic Underground*. New York: Academic Press. pp. 363-385.

[2]. Richards, Norvin. 2001. An idiomatic argument for lexical decomposition. *Linguistic Inquiry* 32: 183-192.

[3]. McCawley, James. 1979. On identifying the remains of deceased clauses. In J. D. McCawley *Adverbs, vowels, and other objects of wonder*. Chicago: University of Chicago Press.
